# Modern finance through quantum computing—A systematic literature review

**DOI:** 10.1371/journal.pone.0304317

**Published:** 2024-07-18

**Authors:** Liliana Bunescu, Andreea Mădălina Vârtei

**Affiliations:** Faculty of Economic Sciences, Lucian Blaga University of Sibiu, Sibiu, Romania; Birla Institute of Technology & Science Pilani - Dubai Campus, UNITED ARAB EMIRATES

## Abstract

Human intellectual restlessness originates from the need for knowledge of the modern world. The financial world is struggling to prototype accurate and fast data at low risk. The quantum approach to finance can support this desire. The goal of this paper is to provide a comprehensive review of the literature on how quantum computing can be used in finance. This research aims to expose an architecture of the state of the art in quantum finance. In terms of methodology, the PSALSAR framework was used to conduct this systematic literature review. The selection procedure followed the PRISMA guidelines and was applied in two databases (Web of Science and Scopus) without time limit. In total, 94 out of 1646 articles were included for data extraction and assessment of content evaluation covering the period 2001–2023. The current review of quantum finance literature is structured around the following themes: journals, research methods, tested data series, research topics in quantum finance, and future research directions. Within the financial sector, quantum computing is used in three main areas: simulation, optimization, and machine learning. These areas are supported by algorithms that have been created in recent years. Finally, we propose to highlight the benefits and the applications of quantum finance and to stimulate the interest in the future prospects of the debates.

## 1. Introduction

The study of quantum finance is still in its infancy. Quantum finance studies the application of quantum computing and quantum information theory to financial and economic problems. It analyzes financial markets and makes investment decisions using mathematical, statistical, and computational techniques. This field uses high-performance technology, complex mathematical models, and algorithms to help identify trends and gain a competitive advantage in making investment decisions.

Although the topic of quantum finance is still in its infancy, interest has been generated by its potential to address some of the issues facing the financial sector, such as optimizing portfolio allocation, enhancing risk management, and creating more precise financial models [1]. The term "quantum finance" refers to the way that researchers from various disciplines, including finance, computer science, quantum computing, and quantum physics, are working together to solve these issues [2].

In the beginning, modern finance theory was built on two pillars: the application of the notion of Brownian motion to stock prices that move randomly and the mathematical expression for rational judgment and decision-making. Modern finance theory was created to solve the conundrum of random market prices [3]. Today, we are facing an era of digital transformation and a new challenge in what modern finance means. The development of modern finance is due to the improvement of automation, the emergence of optimization models in operational activities, the growth of analytical financial thinking, adding value to businesses, and the expansion of digital skills for employees. Skill sets have evolved, and decision-making has become more proactive, so finance organizations have had to adapt to be more competitive in a changing financial market. Modern finance requires maturity in digital, cloud-based big data management solutions to improve financial activity, from reporting to forecasting. Modern finance means that finance can adapt to technology, is growth-oriented, seeks to manage current and future risks, is prepared to make quick decisions, and has some cost awareness. The current roadmap for modern finance is based on two pillars: machine learning and artificial intelligence. In this context, the objective is to identify how quantum computing can be used in the evolution of modern finance.

Quantum computing has the potential to be used in the financial industry to tackle difficult optimization issues, such as the portfolio optimization problem, to optimize predicted returns while minimizing market risks [4]. The process of optimizing a portfolio includes choosing the best mix of assets to generate the desired level of return with the least amount of risk. Using quantum algorithms, quantum finance can offer more efficient portfolio optimization techniques. Classical risk management techniques rely on probabilistic models, which hold that past data can be used to predict future outcomes. However, these models may not be accurate in extremely volatile or unpredict figure markets. Using quantum algorithms that can handle more complex data and consider numerous potential outcomes at once, quantum finance can offer more accurate risk evaluations.

Quantum finance also has the potential to reveal hidden patterns in complex financial data and serve other crucial unmet needs in the financial sector [5]. Pricing models are used to evaluate financial derivatives like options, futures, and swaps. By using quantum algorithms that can more precisely recreate the behavior of financial markets, quantum finance can offer more accurate pricing models. Another potential application of quantum computing in finance is to speed up Monte Carlo simulations, which are extensively utilized in finance for risk management and pricing.

Quantum board in modern finance is to perform calculations that are beyond the capabilities of classical computers, such as simulating complex financial systems and optimizing large portfolios. Computational finance entails the design and implementation of advanced technologies like machine learning, artificial intelligence, and big data analytics to enhance financial analysis and decision-making processes.

This article’s goal is to review the existing body of literature on quantum finance, summarize the results, and propose an integrative framework that covers the potential uses of quantum computing in the financial sector going forward. This study provides a systematic literature review (SLR) quidded by three research directions: (a) the development of the quantum finance field, (b) the state of expertise in quantum finance research today, and (c) possibilities for the future of quantum finance. By this review, we expect to highlight the benefits and applications of quantum finance, increasing the interest in future debates’ perspectives.

The added value of the study can be approached in two ways. First, this paper presents a perspective on the current state of quantum finance research. Second, this paper contributes to a better understanding of this innovative concept and creates a framework for future research in conceptualizations and quantum finance systems development. Through this work, we can pinpoint areas for future study that need further theories, models, and experimental tests. Our systematic review becomes valuable because it increases the statistical power for the analysis of a particular group of papers studying quantum finance. Our research answers questions that have already been investigated by several similar but independent studies of quantum finance. It brings together, in a collective framework, all concepts on the use of quantum computing in finance that have been studied by previous researchers. The uniqueness of the research is explained by the originality of the search strategy of the articles in the sample, by the innovative nature of the adaptation of the use of framework tools in finance (PSALSAR, PICOC), and, finally, by the originality of the presentation of the results obtained.

The format of this article is as follows: the literature review, in Section 2, presents some of the current approaches to the quantum finance field; Sections 3 and 4 highlight the research methodology and the results; the development steps of quantum finance and some specific applications of quantum finance are presented in Section 5, and finally, the article concludes with a summary of key points.

## 2. Literature review

Quantum computers have the potential to revolutionize any industry, including the financial sector, because they can collect all existing and non-existent data in a very brief amount of time to provide the investor with supreme strategy. In this section, distinct perspectives on quantum finance systems will be presented using the specialized literature over the past two decades.

In the early 2000s, quantum theory combined with game theory methods was seen as a possible tool for a deeper understanding of financial phenomena. Quantum finance is an area that can be explored through game theory, where the problem-solving space can be occupied by information beyond our current knowledge, implying the use of calculations from the field of quantum computing. This approach to quantum finance is based on the paradigm that implies that market games are being played by quantum computers using quantum rules [6]. Schaden M. (2002) exposed the paradigm of quantum theory, where the probability of a negative outcome and a non-negative outcome occurring at the same time is fluctuating and indifferent in the financial market because stochastic regions do not have empirical results [7].

One approach to evaluating the well-being of a financial sector is to investigate its level of interconnectedness with other domains, such as the quantum realm [8]. A model of price fluctuations was created using the quantum evolution of physical particles. Drawing on insights from fields such as medicine, geophysics, and radio astronomy, it is possible to speculate about the trajectory of the supply and demand curves in finance [9].

In 2006, a portfolio management model was proposed by rebalancing it periodically for profit maximization and risk minimization. This stochastic financial optimization follows the differences between the Quantum-behaved Particle Swarm Optimization (QPSO) Algorithm model and the classical models to see how they react to the multi-stage portfolio optimization problem [10]. Chang B.R. and Tsai H.F. (2006) proved that adaptive support vector regression (ASVR) applied to the forecast of time series is superior to the other traditional prediction methods, but its prediction accuracy is affected by time-series volatility. In their perspective, incorporating the nonlinear generalized autoregressive conditional heteroscedasticity (NGARCH) model into ASVR deals with the volatility problem and forecasts values in a better way [11].

Research on quantum-like modeling of financial processes is well suited for the quantum finance system’s architecture. This zone is characterized by all previously released information that has an impact on price, as well as any subsequently released information that impacts asset prices at random [12]. In 2008, quantum finance was approached by highlighting a replica quantum-inspired evolutionary algorithm (QIEA), an improvement for the estimation of distribution algorithms (EDAs). This model followed the measurement of volatility for benchmark financial problems with non-linear components and their rate of optimization [13]. In the medium term, quantum finance manifested a new perspective in quantum finance interest rates. More in detail, the space between interest rate and debts is usually called `random noise`or `quantum field`. To mark this zone, a combination was applied between two theories, specifically, the bond forward interest rates, which is a linear theory, and the Libor Market Model, which is a nonlinear theory for understanding the impacts of their types of volatility [2]. Nakayama Y. (2009) proposed novel applications of non-relativistic gauge/gravity correspondence between Reggeon field theory and nonlinear quantum finance. The researcher concludes that there is currently no theoretical foundation that the gravity description is suitable for describing the financial market, but he proposes a solvable model from a completely different perspective [14].

A new interference of understanding the road of quantum financial analysis was introduced in 2010, when the researchers attempted to analyze the dove-hawk relationship in the context of economics, specifically the relationship between investors and the market during crises, using the theory of quantum games. Their findings suggest that with an abundant source of information, it may be possible to decrease the aggressive behavior of investors which can lead to the collapse of the capital market [15]. According to Dai and Zhang (2011), quantum financial forecasting involves the modeling of interest rates and the establishment of prices by using the capacity of the quantum channel independent of Radon-Nikodym variations, which involves the transmission of information to a quantum channel, without the need for prior knowledge of it [16]. Quantum computing presents a potential solution for quantifying or resolving uncertainty in financial market movements [17]. Financial volatility risk and its relation to a business cycle time were addressed by Goncalves C.P. in 2013. His perspective supposes a multiple-round evolutionary quantum game equilibrium related to financial returns and risk dynamics. The model tests real financial volatility data [18].

Quantum economics highlights that quantum theory is a new possibility for understanding the process of the financial market for applying financial arbitrage which represents the risk-free profit or risk-free rate of return. A pattern for this process is found in the return of government bonds because these suppose a certain and sure gain upon maturity of the coupon [19]. Kleinert H. (2014) modeled he degree of occurrence of the Black-Swan phenomenon using quantum modeling [20]. Nastasiuk V.A. (2015) makes a quantum description of financial markets and shows how various models of financial markets are mapped to quantum mechanics [21]. The quantum theory of securities prices points out the probability that a certain return will occur as a reflection of the interference between time and the price value [22].

The development of quantum algorithms that utilize machine learning to analyze stock price performance was discussed by Gao and Chen (2017). These algorithms describe price movements as quantum particles due to the wave fluctuations they record [23]. In 2018, the emphasis fell on developing an algorithm that establishes the best risk-return trade-off curve and permits selection from the best portfolio, where time is quantified as the lowest parameter recorded in the quantum calculation [24]. The interest zone for researchers in 2019 regarding quantum financial analysis was described through models which have three main assignments forecasting financial crashes, controlling the behavior of investors in bear or bull markets, and maintaining financial balance [25]. Research published by Petrenko, and his colleague provides exploratory research on the impact of quantum computing threat on routing and switching IT infrastructure. Their study on large-size financial organizations shows that organizations are facing an increasing risk of potential security threats even if quantum computing technology is developed. The threats affect the existing encrypted data and secured transmission channels [26].

In 2020, the key to modern finance through quantum computing was focused on the impact of the fusion of three key artificial intelligence (AI) tools used in quantum finance: genetic algorithms (GAs) for trading strategy optimization, fuzzy logics (FLs) for fuzzy and inexact financial modeling, and artificial neural networks (ANNs) for machine learning, specifically biological neural models and time series prediction [27]. In the opinion of Lee, the quantum finance system spotlights the behavioral theory branched into the chaos theory and the factual theory in the financial markets which are viewed in the particle-wave duality of the quantum theory. This proposes that the two theories take computational form with the help of nonlinear equations.

During this time, the purpose of quantum theory is to discover the new frontier of finance which involves more intelligent forecasting and trading systems than available through better pricing of financial instruments which can improve the hedging strategies [5]. The evolution of quantum finance software using the Python programming language was unveiled by Wang and Lee. This software promotes the creation of a library of quantum finance calculators [28]. The quantum financial networks know progress for financial risk measurements concerning cryptocurrencies. Faris M. and his colleagues (2023) found evidence of a phenomenon using non-homogeneous data and non-linear mathematical assumptions in testing how dependent and independent variables are related to one another. Specifically, the study focused on the use of Hamiltonian financial quantum material in transactions related to cryptocurrency prices, where the quantum predictor of the price of crypto assets offered a 45% possibility of picking a specular price [29]. A study written by Wilkens S. and Moorhouse J. in 2023 investigates the feasibility of implementing real-world risk measurement applications on quantum computers. Their work contributes to a feasibility analysis of running realistic market risk and counterparty-credit risk applications on quantum devices [30].

All in all, the quantum finance system is distinguished as a mechanism that helps the financial sector to have high performance without traces of high volatility or unpredictability. Hence, with the help of a quantum finance system, the maximum yield of the optimization portfolio and minimum fee risk management are performed more efficiently in less time.

Regarding previous literature reviews in the quantum finance area, we identified several papers that focus on presenting the state of the art in using quantum computing in finance. Gomez A. et al (2022) studied recent advances in quantum computing applied to derivative pricing and the computation of risk estimators [31]. Naik A. et al (2023) have done a review of quantum computing in finance from various perspectives. They concentrated on using quantum computing for blockchain technology [32]. Orús R. et al (2019) examined quantum optimization algorithms and demonstrated how credit scoring, portfolio optimization, and arbitrage opportunity detection may be done using quantum annealers [33]. Tang Y. et al (2019) reviewed quantum optimization and demonstrated how financial issues can be resolved using quantum adiabatic computation [34]. The exemplified papers reveal review aspects of the quantum finance field, but they do not use a systematic literature review framework such as to be replicated or to reduce subjectivity and bias.

## 3. Research methodology

A systematic literature review (SLR) is a rigorous and all-encompassing research approach utilized to discern, assess, and integrate all pertinent studies and publications about a specific research question or topic. According to Baris B. et al (2017), a systematic literature review is an empirical approach employed to extract valuable insights from the vast body of existing research literature [35]. The concept of a systematic literature review was initially developed within the medical field, but it has since evolved to address the unique needs of diverse disciplines. Its main objective is to improve research synthesis by employing a systematic, transparent, and reproducible approach to conducting literature reviews. The process entails various essential steps, such as creating a review protocol, identifying relevant databases, devising appropriate search terms, defining inclusion and exclusion criteria, analyzing the collected data, and finally, synthesizing the findings [36]. This review method is widely employed in academic, scientific, and evidence-based disciplines to furnish an impartial and well-organized synopsis of the existing knowledge on a particular subject. The robustness of a systematic literature review lies in its transparent and reproducible procedures, ensuring that all pertinent studies are considered, minimizing bias, and bolstering the dependability of the synthesized findings. A systematic literature review instills greater confidence in the results obtained, facilitating replication of studies and verification of findings among the scientific community. As a result, systematic literature review continues to be a valuable tool for researchers, driving scientific progress and informed decision-making across disciplines [37].

The method used for conducting the current systematic literature review in quantum finance is PSALSAR developed by Mengist W., Soromessa T., and Legese G. in 2020. PSALSAR is a new method that uses 6 steps for conducting systematic literature reviews, there are two steps added to the usual method SALSA (Search, Appraisal, Synthesis, and Analysis). The new steps are Research Protocol (P) and Reporting Results (R). A clear, portable, and reproducible process for conducting systematic reviews is the PSALSAR approach. It is beneficial to evaluate the literature review’s quantitative and qualitative content analysis [38]. Table 1 presents the steps that were followed for this literature review on quantum finance.

**Table 1:** PSALSAR framework for systematic literature reviews.

STEPS	OUTCOMES	METHODS
**1. PROTOCOL**	Defined study scope	Research question, PICOC framework
**2. SEARCH**	Define the search strategy Search studies	Searching strings Search databases
**3. APPRAISAL**	Selecting studies Quality assessment of studies	Define inclusions and exclusions criteria Quality criteria
**4. SYNTHESIS**	Extract data Categorize data	Extraction template Sort the information according to the iterative definition into categories to make it suitable for additional analysis.
**5. ANALYSIS**	Data analysis Results and discussions Conclusions	Organization of the data into quantitative categories, descriptions, and narrative analysis Display the trend, point out gaps, and compare the results based on the analysis Generating conclusions and advice
**6. REPORT**	Report writing Journal article production	PRISMA methodology Summarizing the report’s findings for a wider audience

The purpose of this systematic review is to produce a comprehensive overview of published peer-reviewed papers about quantum finance. The study aimed to answer the following questions: What are the most recent developments in quantum finance? What is the current state of quantum financial research? What are the future challenges of research on quantum finance? With the help of these questions, we can conduct a literature review that provides a thorough analysis of the contribution of both older and more recent literature in shaping the development of quantum research in finance. The search protocol was done by using the PICOC tool, as can be seen in Table 2.

**Table 2:** PICOC framework for the research scope of quantum finance SLR.

Concept	Quantum finance SRL application
Population	The scientific research work deals with the application of quantum computing and quantum information theory to financial and economic problems.
Intervention	Possibilities to use quantum computing in the financial field (e.g. use of quantum algorithms for behavioral financial markets, use of quantum finance for pricing models, for the trajectory of the supply and demand curves in finance, the design of quantum finance system, the modeling of interest rates, the establishment of prices by quantum financial forecasting, the evolution of quantum finance software, trading strategy optimization, risk management, etc.)
Comparison	Identifying strengths and weaknesses of using quantum theories in finance. Identifying opportunities and threats in using quantum theories for the financial market.
Outcome(s)	Assessment of knowledge and gaps in the selected publications about quantum finance. The most or the least aspects studied about using quantum computing in finance. Perks and limitations of quantum finance. Synthesis of methods used in quantum finance studies.
Context	Trends of quantum finance research, challenges and gaps in quantum finance application to solve complex problems (e.g. optimization, pricing models, risk management, financial forecasting, artificial intelligence impact, etc.) Geographical distribution of existing studies.

The search was performed in two well-known and reliable databases, Scopus, and Web of Science, in July 2023. The objective is to get much more yield and more accurate and comprehensive results. The search was done by using the search terms “*quantum”* and “financ*” in the title, abstract, and keywords. The terms were selected to capture the widest possible array of research literature about quantum finance. The year of publishing was left unrestricted.

In the appraisal phase, the studies were selected by using inclusion and exclusion criteria, as can be seen in Table 3. The quality assessment was done by screening the papers to find the pertinent articles for the review work. The papers that did not fit the inclusion criteria were excluded after the titles and abstracts of the identified records were reviewed. The full text of the remaining records was then assessed for eligibility.

**Table 3:** Inclusion and exclusion criteria for quantum finance SRL.

Criteria	Decision
The searching terms exist in title, abstract or keywords of the papers.	Inclusion
The papers are published in a peer-reviewed journal or conferences.	Inclusion
The papers are in English.	Inclusion
The papers contain concepts which are relevant to the research objective. The paper provides information about using quantum theory in finance.	Inclusion
Papers that are duplicate, indexed in both databases.	Exclusion
Papers with no abstract available.	Exclusion
Papers that are written in other languages than English.	Exclusion
Papers that are not accessible as full text (they are not open-access).	Exclusion
Grey literature, books, chapters, editorial materials, corrections / erratum, notes, letters, new items, surveys.	Exclusion

2,135 papers were identified using the search terms in the chosen databases, 692 papers in Web of Science, and 1,443 papers in Scopus. 45 papers written in other languages than English were removed. In this review the articles published in journals and conference proceedings were integrated, because they have been reviewed according to a step-by-step review process, thus 444 records were removed. The title screening was performed for 1,646 papers and led to the elimination of 1,420 papers that were not relevant to the research objective of our review. Further, we got 226 papers about quantum finance, of which 102 papers were indexed in Web of Science and 124 papers were indexed in Scopus.

**Fig 1:**
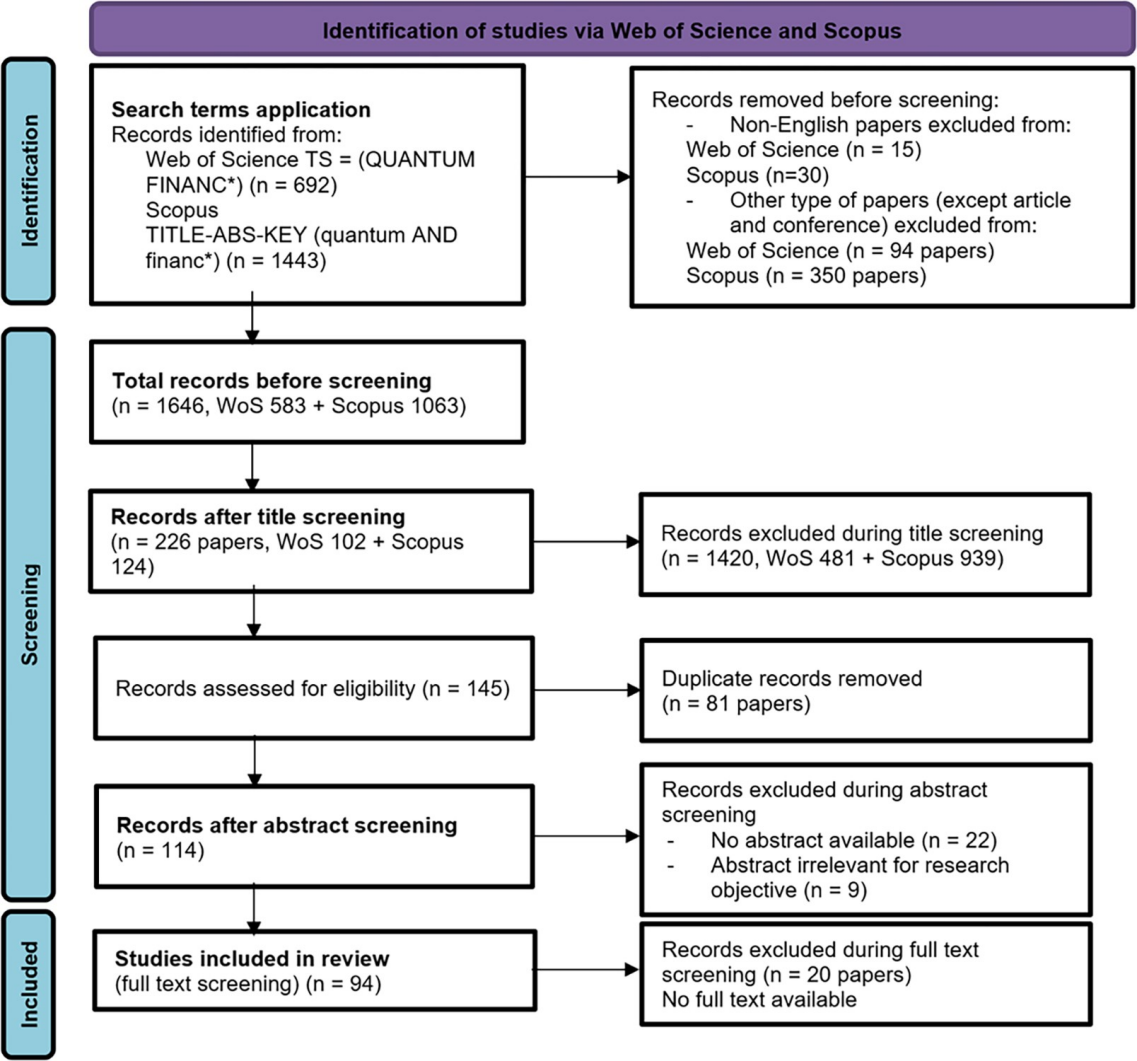
PRISMA Diagram showing the screening process.

At this point, the results were exported from both databases as Excel files (authors, title, journal, year, abstract), and the results were merged and manually deduplicated. 81 papers were removed because they were indexed in both databases. All publications with the same title, author, and publication date in the same year and journal were removed from the sample. The remaining papers (145) were assessed for eligibility. Quality assessment was performed based on two quality assessment (QA) questions: QA1. Is the literature search covering a relevant study on the topic of quantum finance? QA2. Was adequately described the type of using quantum theory in finance in the publication? 31 papers were removed in the abstract screening step to minimize the chance of including non-relevant articles. We manually downloaded the articles as PDF files by using different online search engines to access full-text articles (e.g. ResearchGate, Arxiv, etc). For 20 papers the full–text was not available as open–access, so they were removed. The present systematic literature review on quantum finance has a total of 94 papers which are detailed in the S1 Appendix. The sample manuscripts cover over two decades. These 94 papers were published between 2001 and 2023. The data collection of included full texts was manually done in a structured Excel sheet. Fig 1 is presenting the entire detailed selection process. The data were assessed by two reviewers who worked independently, and the differences that arose were resolved together. Automation tools were not used in the process.

## 4. Results

The current quantum finance literature review is structured around five themes: (a) journals that publish research in quantum finance, (b) research methods that were used to analyze quantum finance, (c) data series tested by quantum finance models or algorithms, (d) research topics in quantum financed that were addressed by researchers and (e) future research direction identified by authors who focused on research in this field.

Regarding the first issue, *Physica A* is the journal where most papers about quantum finance were published (21%). It is followed by *Quantum* Journal (7%) and *Physical Review E* (5%). In Table 4 it can be found the entire list of journals where the research papers about quantum finance can found. It should be added that a large number of papers were published in conference proceedings, such as 17th Conference on the Theory of Quantum Computation, Communication and Cryptography (TQC 2022), International Joint Conference on Neural Networks (2006), IEEE International Conference on Services Computing (SCC 2021), E3S Web of Conferences, IEEE International Conference on Machine Learning Applications (ICMLA 2022), 9th Joint International Conference on Information Sciences (JCIS-06), 29th International Joint Conference on Artificial Intelligence (IJCAI-20), etc.

**Table 4:** Number of articles about quantum finance per journal and number of publications per year.

Journal name	Number of publications	2001–2005	2006–2010	2011–2015	2016–2020	2021—present
A Letters Journal Exploring the Frontiers of Physics	2			1	1	
Acta Physica Polonica A	1				1	
Acta Physica Polonica B	1	1				
Archives of Computational Methods in Engineering	1					1
Beyond Traditional Probabilistic Methods in Economics	1				1	
Computational Economics	1					1
Computational Intelligence	1		1			
Computers and Mathematics with Applications	2			1	1	
Electronic Journal of Theoretical Physics	1		1			
Entropy	2					2
Expert Systems with Applications	2			1		1
Fluctuation and Noise Letters	1					1
Information Sciences	1		1			
International Journal of Electrical and Computer Engineering (IJECE)	1					1
International Journal of Modern Physics A	1		1			
International Journal of Pure and Applied Mathematics	1			1		
International Review of Applied Economics	1					1
Investment Management and Financial Innovations	1				1	
Journal of Financial Regulation and Compliance	1			1		
Journal of Information Security and Applications	1				1	
Journal of Payments Strategy & Systems	1				1	
Journal of Physics	3		1	1		1
Machine Learning: Science and Technology	1				1	
Mathematics	1					1
Neurocomputing	1		1			
Physica A	20	1	6	5	7	1
Physical Review A	2				2	
Physical Review E	5		5			
Physical Review Research	2				1	1
Physics Letters A	1			1		
Physics World	1	1				
Quantitative Finance	1	1				
Quantum	7		2	2		3
Quantum Computing in Econometrics and Quantum Economics and Related Topics	1				1	
Quantum Information Processing	3					3
Quantum Science and Technology	2				1	1
Reviews in Physics	1				1	
Scientific Programming	1					1
Scientific Reports	1					1
Service Oriented Computing and Applications	1					1
SIAM Journal on Financial Mathematics	1				1	
Studies in Computational Intelligence	1				1	
Conference Proceedings	12		4	2	1	5
**Total**	**94**	**4**	**23**	**16**	**24**	**27**

From a temporal perspective, quantum finance has been intensively studied in the last two decades. The research had been intensified in the last 8 years when 54% of the publications in the sample were issued.

Table 5 centralizes the research methods applied by the researchers in the quantum finance study. Mathematical models and algorithms are the core research paths used to identify how quantum computing can be used in finance issues. 89% of studies are based on creating hybrid and quantum algorithms to create different means of using the quantum theory in the field of private finance. The number of 7 publications contains issues about the conceptual approach of quantum finance. To these, are added 2 papers with experimental tests and another one with interviews. No paper with a systematic literature review was identified in the sample.

**Table 5:** Research methods used in quantum finance over time.

Research method	Number of publications	2001–2005	2006–2010	2011–2015	2016–2020	2021—present
Mathematical model / Algorithm	84	2 2	24	14	21	23
Conceptual / Theoretical	7	2		2	2	1
Experimental test	2					2
Interview	1					1
**Total**	**94**	**4**	**24**	**16**	**23**	**27**

Table 6 centralizes the data series which were tested by quantum or hybrid models. First, it should be noticed that only 21 publications on quantum finance, out of 94 publications, contained information about testing the mathematical algorithms approached in the research by using real financial data. The most tested data series are represented by stock exchange indexes, the most well-known from the United States, India, China, the United Kingdom, Japan, Taiwan, and South Korea. On the second rank, there are data series represented by stocks and options. They are used in the quantum finance model for price forecasting or portfolio quantum optimization. Commodities, currency pairs, and interest rates were used to provide real data series tested in quantum algorithms.

**Table 6:** Analysis of types of data series tested by quantum finance models.

Type of data series	Study site	Scale	No. of pubs	2001–2005	2006–2010	2011–2015	2016–2020	2021—present
Securities	347 stocks	NYSE (USA)	1				1	
	Apple, Amazon, Boeing, Bank of America, British Petroleum, Caterpillar, Cisco, General Electric, Alphabet, Home Depot, International Business Machines, Juniper Networks, JPMorgan, Nordstrom, Coca Cola, Manulife Financial, 3M, Merck, Microsoft, Oracle, Pfizer, Royal Dutch Shell, Toronto Dominion, Walmart	NASDAQ (USA)	1					1
	Applied Materials, Exxon Mobil, General Electric, Hewlett Packard	NASDAQ (USA)	1		1			
	Exxon Mobil, Hewlett-Packard	NASDAQ (USA)	1		1			
	Tesla, Alnylam Pharmaceuticals	NASDAQ (USA)	1				1	
	Swaptions	US, Singapore, Malaysia	1				1	
Indexes	CNX Nifty	National Stock Exchange (India)	1				1	
	CSI 300, SSE 50, SSE 180, SZSE Index, SSEC Index, GEM Index	Shanghai Stock Exchange (China)	1					1
	NASDAQ Index	NASDAQ—USA	1		1			
	New York D.J. industrials index, London FTSE-100 index, Tokyo Nikkei index, Taipei TAIEX index	USA, UK, Taiwan, Japan	1		1			
	New York D.J. Industrials Index, Taipei TAIEX Index, Tokyo Nikkei Index, Seoul Composite Index	USA, Taiwan, Japan, South Korea	1		1			
	Nifty 50	India	1					1
	S&P 100	NYSE (USA)	1		1			
	S&P 500	NYSE—USA	1				1	
Commodities	Oil and gold market	-	1			1		
Interest rates	LIBOR, EURIBOR	-	1		1			
Currency pairs	EUR/USD, GBP/USD	-	1				1	
Other	Kaggle, financial data from companies, financial data from mobile phones etc.)	-	4					4
**Total**	-	-	**21**	**0**	**7**	**1**	**6**	**7**

To meet the objectives of the present systematic literature review, the quantum finance research topics were assessed and centralized in Table 7. By manual scanning of articles’ full text, approximately 90 approaches were identified and used to develop this field. It should be noted that some papers refer to one quantum finance approach, but many papers address more than one quantum finance approach. The heterogeneity of concepts used to study quantum finance has led to the difficulty in identifying some grouping keys for all these quantum finance research topics. However, we synthesized all the quantum finance approaches into five groups: (1) quantum finance used to describe financial markets; (2) quantum finance models or algorithms used to describe financial derivatives; (3) mathematical concepts used in quantum finance, including different formula, equations, conditions; (4) quantum techniques used for optimization, and (5) concepts from quantum theory used in finance.

**Table 7:** Research themes in quantum finance.

Quantum finance approaches		Pubs	Authors
**1**	**Financial markets (risk, crisis, time series predictions)**	**43**	Aboussalah, AM; Chi, C; Lee, CG (2023); Alcazar, J; Leyton-Ortega, V; Perdomo-Ortiz, A (2020); An, D et al (2021); Araújo R.D.A., De Oliveira A.L.I., Soares S.C.B. (2010a); Araujo, R.D.A.; de Oliveira, A.L.I.; Soares, S.C.B. (2010b); Arraut, I; Au, A; Tse, ACB (2020); Baaquie, B.E. (2007); Bai, L et al (2020); Barad, G (2012); Chang B.R., Tsai H.F. (2006a); Chang, B.R.; Tsai, HF (2006b); Chang, BR; Tsai, HF (2009); Cruz, P; Cruz, H (2020); Ding, YC et al (2023); Gomez, A et al (2022); Goncalves, CP (2011); Griffin, P; Sampat, R (2021); Hanauske, M et al (2010); Henkel, C (2017); Hwang J.H. (2015); Kaneko, K et al (2021); Kim, MJ et al (2011); Manjunath C., Marimuthu B., Ghosh B. (2023); Mugel S., Lizaso E., Orús R. (2020); Nastasiuk, VA (2014); Nastasiuk, VA (2015); Orús R., Mugel S., Lizaso E. (2019); Orus, R; Mugel, S; Lizaso, E (2018); Paquet, E; Soleymani, F (2022); Racorean O. (2013); Romero J.M., Lavana U., Miranda E.M. (2014); Schaden, M (2002); Wang, CF; Yang, YK; Xu, LL; Wong, A (2023); Wilkens, S; Moorhouse, J (2023);
	Financial Time Series Forecasting	14	
	Volatility models, price volatility, The Greeks, volatility clustering effect, chaotic volatility	10	
	Systematic risk, risk management, value at risk (VaR), financial risk, risk analysis	7	
	Financial shock, financial crisis, prediction of financial crashes	5	
	NGARCH Composite Model (nonlinear type of generalized autoregressive	2	
	Dynamic Time Warping (DTW)	2	
	Other topics (Random Walk Dilemma, Time Phase Distortions Adjustment, Generative models)	3	
**2**	**Models for financial derivatives**	**65**	An, D et al (2021); Araújo R.D.A., De Oliveira A.L.I., Soares S.C.B. (2010a); Araujo, R.D.A.; de Oliveira, A.L.I.; Soares, S.C.B. (2010b); Baaquie B.E. (2008a); Baaquie, B.E. (2018); Baaquie, B.E. (2007); Baaquie, B.E. (2013); Baaquie, B.E. (2010); Baaquie, B.E. (2009); Baaquie, B.E. (2008b); Baaquie, BE; Du, X; Tang, P; Cao, Y (2014); Baaquie, BE; Liang, C (2007a); Baaquie, BE; Liang, C (2007b); Baaquie, BE; Pan, T (2011); Baaquie, BE; Tang, P (2012); Baaquie, BE; Yang, C (2009); Baaquie, BE; Yu, M; Bhanap, J (2018); Bagheri, A; Peyhani, HM; Akbari, M (2014); Barad, G (2012); Darbyshire, P (2005); Doriguello J.F. et al (2022); Fernandez-Lorenzo, S; Porras, D; Garcia-Ripoll, JJ (2021); Fontanela, F; Jacquier, A; Oumgari, M (2019); Gomez, A et al (2022); Hellstem, G (2021); Ingber L. (2015); Khrennikov, A (2007); Kim, MJ et al (2011); Manjunath C., Marimuthu B., Ghosh B. (2023); Martin, A et al (2019); Melnyk S.I., Tuluzov I.G. (2008); Orús R., Mugel S., Lizaso E. (2019); Paquet, E; Soleymani, F (2022); Rebentrost, P; Gupt, B; Bromley, TR (2018); Romero, JM; Miranda, EM; Lavana, U (2014); Stamatopoulos, N et al (2021); Wilkens, S; Moorhouse, J (2023);
	Interest rate options, interest rate swap, swap options, Libor, forward interest rate, accrual swap, caplet	13	
	Hybrid model	11	
	European options on coupon bonds, index-linked coupon bonds, American options on coupon bond	10	
	Financial Derivatives	9	
	Option pricing model	8	
	Heath, Jarrow and Morton (HJM) model	3	
	Barrier options	3	
	BGM–Jamshidian model	2	
	Black’s caplet formula	2	
	Other topics (sukuk, options on stocks, automatic differentiation, options on foreign exchange)	4	
**3**	**Financial mathematics**	**68**	An, D et al (2021); Arraut, I; Au, A; Tse, ACB (2020); Arraut, I; Marques, JAL; Gomes, S (2021); Baaquie, B.E. (2018); Baaquie, B.E. (2009); Baaquie, B.E. (2008b); Baaquie, BE; Du, X; Tang, P; Cao, Y (2014); Bagheri, A; Peyhani, HM; Akbari, M (2014); Barad, G (2012); Biesner, D et al (2022); Cruz, P; Cruz, H (2020); Darbyshire, P (2005); Doriguello J.F. et al (2022); Farinelli S., Takada H. (2022); Fontanela, F; Jacquier, A; Oumgari, M (2019); Gomez, A et al (2022); Haven E. (2007); Henkel, C (2017); Kaneko, K et al (2021); Martin, A et al (2019); Melnyk S.I., Tuluzov I.G. (2008); Nastasiuk, VA (2014); Nastasiuk, VA (2015); Orús R., Mugel S., Lizaso E. (2019); Orus, R; Mugel, S; Lizaso, E (2018); Racorean O. (2013); Rebentrost, P; Gupt, B; Bromley, TR (2018); Romero J.M., Lavana U., Miranda E.M. (2014); Romero, JM; Miranda, EM; Lavana, U (2014); Schaden, M (2002); Stamatopoulos, N et al (2021); Tahmasebi, F et al (2015); Tang, YH et al (2022); Yaghobipour, S; Yarahmadi, M (2018); Yaghobipour, S; Yarahmadi, M (2020); Yeşiltaş Ö. (2023)
	Black—Scholes equations, Black-Scholes Quantum formula	13	
	Hamiltonian approach	13	
	Monte Carlo methods	12	
	Schrodinger Equation	8	
	Stochastic differential equations (SDEs), stochastic Taylor expansion, Ito calculus	6	
	Martingale condition	3	
	Merton-Garman equations	2	
	Spontaneous symmetry breaking	2	
	Other topics (fractals, Fisher information approach, Hilbert Space, Poisson Jump Process, Hull-White model, Wavelet Transform (WT), Hopf algebra, Hamilton–Jacobi–Bellman (HJB) equation, subset sum)	9	
**4**	**Quantum optimization techniques**	**20**	Aboussalah, AM; Chi, C; Lee, CG (2023); Alcazar, J; Leyton-Ortega, V; Perdomo-Ortiz, A (2020); Bagheri, A; Peyhani, HM; Akbari, M (2014); Biesner, D et al (2022); Ding, YC et al (2023); Fernandez-Lorenzo, S; Porras, D; Garcia-Ripoll, JJ (2021); Griffin, P; Sampat, R (2021); Mugel S., Lizaso E., Orús R. (2020); Nakaji, K et al (2021); Orús R., Mugel S., Lizaso E. (2019); Orus, R; Mugel, S; Lizaso, E (2018); Pan, WT et al (2021); Piotrowski, EW; Sladkowski, J (2001); Qiu Y., Liu R., Lee R.S.T. (2021); Sun, J; Xu, WB; Fang, W (2010); Tang, YH et al (2022); Yaghobipour, S; Yarahmadi, M (2018); Yaghobipour, S; Yarahmadi, M (2020);
	Optimization model, quadratic unconstrained binary optimization (QUBO), Fruit Fly Optimization Algorithm (QFOA), Bee Colony Optimization Algorithm (QABC), Quantum-behaved Particle Swarm Optimization (QPSO), Ant Colony Optimization (QACO), a higher-order unconstrained binary optimization (HUBO)	10	
	Portofolio optimization	9	
	Variational quantum algorithms (VQAs)	1	
**5**	**Quantum mechanics / theory**	**110**	Aboussalah, AM; Chi, C; Lee, CG (2023); Alcazar, J; Leyton-Ortega, V; Perdomo-Ortiz, A (2020); Araújo R.D.A., De Oliveira A.L.I., Soares S.C.B. (2010a); Araujo, R.D.A.; de Oliveira, A.L.I.; Soares, S.C.B. (2010b); Baaquie, B.E. (2007); Baaquie, BE; Liang, C (2007a); Baaquie, BE; Liang, C (2007b); Bagheri, A; Peyhani, HM; Akbari, M (2014); Bai, L et al (2020); Biesner, D et al (2022); Chang B.R., Tsai H.F. (2006a); Chang, B.R.; Tsai, HF (2006b); Chang, BR; Tsai, HF (2009); Chen, CM; Tso, GKF; He, KJ (2023); Choustova O. (2007a); Choustova O. (2007b); Choustova, O (2009); Choustova, OA (2007c); Covers O., Doeland M. (2020); Coyle, B et al (2020); Cruz, P; Cruz, H (2020); Darbyshire, P (2005); Ding, YC et al (2023); Dupoyet, B; Fiebig, HR; Musgrove, DP (2010); Farinelli S., Takada H. (2022); Feng, XN et al (2022); Ghosh B., Kozarevic E. (2018); Gomez, A et al (2022); Goncalves, CP (2011); Griffin, P; Sampat, R (2021); Hanauske, M et al (2010); Haven E. (2007); Haven E. (2019); Hellstem, G (2021); Henkel, C (2017); Ingber L. (2015); Khrennikov, A (2007); Khrennikova P. (2019); Kim, MJ et al (2011); Mancilla, J; Pere, C (2022); Manjunath C., Marimuthu B., Ghosh B. (2023); Martin, A et al (2019); Nakaji, K et al (2021); Nakayama, Y (2009); Nastasiuk, VA (2014); Orús R., Mugel S., Lizaso E. (2019); Orus, R; Mugel, S; Lizaso, E (2018); Paquet, E; Soleymani, F (2022); Petrenko, K; Mashatan, A; Shirazi, F (2019); Piotrowski, EW; Sladkowski, J (2004); Piotrowski, EW; Sladkowski, J (2001); Pistoia, M et al (2021); Qiu Y., Liu R., Lee R.S.T. (2021); Sarkissian, J (2020); Schaden, M (2002); Singh, S; Subrahmanya, MHB (2021); Stamatopoulos, N et al (2021); Tahmasebi, F et al (2015); Tang, YH et al (2022); Wang, CF; Yang, YK; Xu, LL; Wong, A (2023); Wilkens, S; Moorhouse, J (2023); Yaghobipour, S; Yarahmadi, M (2018); Yeşiltaş Ö. (2023)
	Behavioral financial model, artificial neural network, quantum neural network, Hopfield networks, mobile behavior	14	
	Quantum mechanics	12	
	Quantum machine learning	9	
	Noisy intermediate-scale quantum (NISQ)	7	
	Bohmian Mechanics, pilot wave model	6	
	Quantum game theory, Hawk–dove game	5	
	Bohm-Vigier model, stochastic mechanics	4	
	Quantum probabilistic model, quantum probability	4	
	Restricted Boltzmann machines (RBMs)	3	
	Quantum Inspired Evolutionary Algorithm (QIEA)	3	
	Feynmann diagrams / perturbation	3	
	Quantum minimization	3	
	BPNN-weighted GREY-C3LSP prediction	3	
	Quantum cryptography, cyber security, cryptographyc algorithms	3	
	Approximate Amplitude Encoding, quantum amplitude estimation	3	
	Quantum circuit Born mashines (QCBMs)	2	
	Quibit MultiLayer Perceptron (QuMLP)	2	
	Lattice theory	2	
	D-Wave 2000Q quantum annealer, quantum annealers, D-Wave machine	2	
	Quantum Social Science (QSS), quantum world in social phenomena	2	
	Support Vector Machine (SVM)	2	
	Other topics (Quantum partitioning, Weak-value, Double slit experiment, Adaptive Network-based Fuzzy Inference System (ANFIS), Quantum Entropy, continuous-time quantum walk (CTQW), Tight-binding methods, Gauge invariance, quantum blind signature, Khrennikov Quantum Potential Quantum-like Approach, quantum path-integral, quantum data encoding, Reggeon field theory, Quantum Zeno effect, Deep Reinforcement Learning (DRL), Quantum coupled-wave theory)	16	

Our analysis highlights that research in quantum finance is focused on using and finding applications of a large category of quantum theory concepts in the finance field, particularly in financial markets aspects. Artificial neural networks, quantum mechanics, and quantum machine learning are the most debated issues. To these is added quantum probability, quantum game theory, quantum cryptography, quantum annealers, or quantum social science. Then, the second position belongs to the merger between financial mathematics and quantum physics. Some of the most frequently used mathematical concepts in quantum finance are Black—Scholes equations, the Hamiltonian formula, the Monte Carlo methods, and the Schrodinger equation. The third, and also a large category of research topics in quantum finance goes to option pricing models, in general, or specific categories of options, in particular. The most studied hybrid or quantum algorithms are for interest rate options and interest rate swaps, European and American options on coupon bonds, and other financial derivatives (e.g. barrier options, sukuk, options on stocks, options on foreign exchange pairs). The fourth group of research topics in quantum finance refers to quantum models used to forecast financial time series, quantum models for price volatility, quantum models for risk analysis, and financial shock prediction. The last group of articles about quantum finance includes research on quantum models for portfolio optimization and details about optimization models (i.e. Quadratic Unconstrained Binary Optimization, Quantum-behaved Particle Swarm Optimization, A Higher-Order Unconstrained Binary Optimization, etc.)

Table 8 centralizes the directions of further research that the authors have proposed in the published works. 38% of publications contain references to aspects related to quantum finance particularities that can be detailed and deepened in further research. The future directions of research are particularized at the level of each group of authors, pursuing either the improvement of the proposed quantum models, or their testing. Numerous papers do not refer to future research in quantum finance.

**Table 8:** Future research directions in quantum finance.

Authors	Future research directions
Alcazar, J; Leyton-Ortega, V; Perdomo-Ortiz, A (2020)	expansion of quantum simulations to more qubits while employing target distribution samples as a training set for both classical and quantum models.
An, D; Linden, N; Liu, JP; Montanaro, A; Shao, CP; Wang, JS (2021)	provision of other meaningful characteristics of stochastic processes, finding more practical quantum input-output models for potential applications in finance
Arraut, I; Au, A; Tse, ACB; Segovia, C (2019)	modeling the uncertainties in the prices of the options by using the double-slit approach and the concept of weak-value
Arraut, I; Marques, JAL; Gomes, S (2021)	using symmetry arguments to analyze the flow of information in the stock market and its visualizations
Baaquie, BE (2018)	to research the situation in which the tenor, principal payments, and quantity of the coupon payments are all subject to stochastic variation
Baaquie, BE; Yu, M; Bhanap, J (2018)	to study Malaysian forward interest rates
Bagheri, A; Peyhani, HM; Akbari, M (2014)	to construct a real-time warning system by using the suggested method on shorter timescales.
Biesner, D; Gerlach, T; Sifa, R; Bauckhage, C; Kliem, B (2022)	to create an algorithm for smart auditing software
Chen, CM; Tso, GKF; He, KJ (2023)	optimization directions for their model: other quantum population selection techniques can be tested; the introduction of profit-based measures in their solution; performance and cost tradeoff analysis of the proposed method on many targets or labels scenario
Covers O., Doeland M. (2020)	to deal with the threats associated with quantum computing,
Coyle, B; Henderson, M; Le, JCJ; Kumar, N; Paini, M; Kashefi, E (2020)	to increase Born Machine training efficiency, to increase performance by considering quantum-specific optimizers for the model and training, expanding the range of classical model comparison, and researching ways to divide the classical-quantum resources in the learning process
Cruz, P; Cruz, H (2020)	to study the possibility of having a moving indicator based on a quantum mechanical tool
Doriguello J.F., Luongo A., Bao J., Rebentrost P., Santha M. (2022)	how and when their algorithm can find application in practice
Dupoyet, B; Fiebig, HR; Musgrove, DP (2010)	examination of the lattice characteristics of the model’s parameters, including looking for phase transitions or spontaneous symmetry-breaking
Feng, XN; Wu, HY; Zhou, XL; Yao, Y (2022)	the creation of a quantum blind signature system to address the noise situation and complete the design
Fontanela, F; Jacquier, A; Oumgari, M (2019)	to design an efficient ansatz for more complex financial products, or in the development of an ansatz-free approach
Ghosh B., Kozarevic E. (2018)	utilization by policymakers of the financial Reynolds number as an indicator of market volatility
Gomez, A; Leitao, A; Manzano, A; Musso, D; Nogueiras, MR; Ordonez, G; Vazquez, C (2022)	to discover effective unitary transform-based mathematical representations of payoff functions that can be readily implemented in a quantum circuit, as well as efficient methods for loading probability distributions into quantum registers
Griffin, P; Sampat, R (2021)	to improve quantum hardware, processing speeds, and data volumes that quantum offers
Hellstem, G (2021)	to develop hybrid quantum networks
Henkel, C (2017)	to find solutions of stochastic differential delay equations
Kaneko, K; Miyamoto, K; Takeda, N; Yoshino, K (2021)	to explore the possibility of making quantum algorithm for Monte Carlo more efficient
Khrennikova P. (2019)	to apply the theory of quantum probability’s utility to the financial market considering behavioral anomalies and state-dependent preferences
Mancilla, J; Pere, C (2022)	to expand the method to include additional datasets (with more attributes) in additional domains to establish a benchmark through a broad application
Nakaji, K; Uno, S; Suzuki, Y; Raymond, R; Onodera, T; Tanaka, T; Tezuka, H; Mitsuda, N; Yamamoto, N (2021)	to test their algorithm with a real quantum computing device.
Nastasiuk, VA (2015)	to find analytically solvable equations and use widely available financial data
Orús R., Mugel S., Lizaso E. (2019)	to research the applications of quantum simulators in finance, the impact of quantum cryptography on the security of financial transactions, and how quantum technologies can be relevant to the blockchain and cryptocurrencies
Orus, R; Mugel, S; Lizaso, E (2018)	to identify ways of improving the efficiency and accuracy of their procedure, to deal with other financial equilibrium problems
Pan, WT; Liu, Y; Jiang, H; Chen, YT; Liu, T; Qing, Y; Huang, GH; Li, R (2021)	to suggest additional strategies for improving the four algorithms, including wavelet or chaos theory
Piotrowski, EW; Sladkowski, J (2004)	markets cleared by quantum algorithms (computers)
Pistoia, M; Ahmad, SF; Ajagekar, A; Buts, A; Chakrabarti, S; Herman, D; Hu, SH; Jena, A; Minssen, P; Niroula, P; Rattew, A; Sun, Y; Yalovetzky, R (2021)	enhancing the financial sector with quantum machine learning methods in the NISQ era
Qiu Y., Liu R., Lee R.S.T. (2021)	to develop a multi-agent mechanism
Racorean O. (2013)	to see particle physics effects in the stock market
Rebentrost, P; Gupt, B; Bromley, TR (2018)	investigating the promising advantages of the continuous variable quantum computation setting in a financial context
Sarkissian, J (2020)	understanding financial processes progresses from a basic analogy-based level to a level based on physical nature.
Stamatopoulos, N; Mazzola, G; Woerner, S; Zeng, WJ (2021)	a detailed comparison of the performance between the quantum gradient algorithms and AD methods

The importance of a systematic literature review lies in giving space to the theories that underpin previous research. Considering theoretical grounding, we propose to synthesize the conceptual contribution of prior quantum finance investigations in a systematic literature review, which will be able to strengthen our theoretical base through a close understanding of the conceptual framework within which our paper fits. After the analysis of the theories, models and algorithms proposed by the 94 articles, it is worth mentioning that they can be divided into 6 categories, pursuant to the similar particularities they share: Modeling Techniques (23 manuscripts), Prediction Techniques (22 manuscripts), Theory Application (22 manuscripts), Market Dynamics (9 manuscripts), Risk Management (9 manuscripts), and Financial Algorithms (9 manuscripts). Quantum finance modeling is predominant in the sample, but there are 6 papers where no mathematical model was identified. However, these papers study quantum finance for financial management risk, keeping payments safe by using quantum computing, quantum game theory, or they present theoretical perspective of quantum physics and classical finance. A particular group of papers are addressing to reviewing previous literature on different quantum finance topics such as: research on quantum computing applied to derivatives pricing, development of quantum game theory, quantum optimization, and the utility of quantum theory in finance.

The other papers are handling mathematical models for incorporating quantum physics in finance. A detailed situation can be found in the S2 Appendix, table in which we tried to capture the particularities of each quantum finance model, the problems which are solved by it, the limitations of the models pointed out by their authors, and future challenges for improving the actual quantum finance models. The most explored dimensions of quantum finance are based on financial prediction techniques, the application of financial theory and financial modeling, where the analysis of financial market dynamics, risk management and financial algorithms are in the background and deserve further investigation in the future to be able to track financial market vulnerabilities at the expense of quantum computing in optimizing an efficient prediction model.

The relevance of our scrutiny achieves a framework through the introduction of gaps in the quantum finance literature, which enhances continued progress in studying the area. The shortcomings and inadequacies reported by the researchers highlight: risks and vulnerabilities, adoption, and implementation of quantum technologies in the financial sector, assessing the socio-economic impact of adopting quantum technologies and concepts in finance, exploring, and improving the security and resilience of quantum technologies in the financial system. Also, it is noted the lack of evolutive integration of quantum technologies into traditional financial infrastructure, the use of quantum computing to solve complex problems in finance, the study of the interconnection between technological and human factors in anchoring quantum technology in finance. And one area waiting to be explored is the quantification of quantum theories for prototyping behavioral financial markets.

## 5. Discussions

### (a) Quantum Finance’s Perks

Within the financial sector, quantum computing is utilized in three primary areas: simulation, optimization, and machine learning. These areas are underpinned by algorithms that have been created in recent years. In optimization, quantum technology can improve credit risk classifications, enable precise customer segmentation, and enhance the detection of fraudulent activities.

Cryptography and cybersecurity are areas where quantum computing can be used to develop more secure encryption methods that are resistant to quantum hacking. Encrypting access keys can increase the security of financial information. However, current encryption systems may become vulnerable to the immense computing power of quantum computers. Therefore, the use of quantum computers is expected to lead to the development of new Internet security protocols. The analyzed data also appears to have a high degree of confidentiality. Furthermore, quantum cryptography has the potential to bolster the security of financial transactions and safeguard them against cyberattacks.

Speed and efficiency are the abilities to process complex financial calculations significantly faster than classical computers, quantum computing offers the potential for quicker risk assessment, more efficient trading strategies, and faster fraud detection. The volume of data that is retrieved, stored, and processed within very short time frames is enormous. Quantum computing enables the modeling of financial formulas that accurately predict the value of derivative prices, closely aligning with the fluctuations in the actual market. By expediting the financial optimization, forecasting, and modeling procedures, it is possible to reduce the overall fees of the process over time.

Simultaneously, it aids investors in managing their portfolios by providing highly precise forecasts, thereby minimizing their risks. In quantum investment management, with advanced knowledge of market movements, investors can avoid being swayed by the herd mentality and instead follow a rational, emotion-free approach. However, this reliance on calculated predictions cannot guarantee the success of the entire investment collective, as certain emotional factors cannot be accurately quantified. Investors face significant challenges due to market volatility and uncertainty. Quantum computing can help in developing models that simulate reality, allowing investors to manage these variables more effectively. Additionally, quantum computing can improve the accuracy of financial analysis by enabling the exploration of multiple scenarios and probabilities simultaneously, resulting in more precise predictions of financial outcomes. The computational power of quantum financial modeling possesses the potential to handle exponentially larger datasets compared to classical computers, thus enabling the creation of more intricate and sophisticated financial models. Quantum algorithms can also perform complex mathematical operations in a matter of seconds, enabling the rapid detection of anomalies and patterns in financial data that may have otherwise gone unnoticed.

The concept of quantum superiority refers to the ability of quantum computers to perform intricate computations in a matter of seconds, a task that would require supercomputers several decades to complete. Moreover, quantum computers possess the property of quantum overlap, enabling the simultaneous calculation of multiple financial states. The need for quantum computing is reinforced by the use of specialized algorithms that utilize faster information units called qubits, which provide a significant improvement over classical computer bits. By harnessing these capabilities, it is possible to perform joint computations between artificial intelligence and quantum algorithms, making it possible to find efficient solutions to complex financial problems.

### (b) Rollout of Quantum Finance

Computational financial mapping is the route that can be developed to find solutions by applying quantum computing in finance.

1. *Identify the financial challenges*—A crucial aspect for researchers and professionals in the field is to acknowledge and comprehend the hurdles and complexities that emerge when utilizing quantum computing in finance. By doing so, they can craft innovative solutions and methodologies that tackle these challenges, propelling the development of quantum finance to unprecedented levels.
2. *Designing a quantum algorithm*—The success of applying quantum finance systems depends on the creation of quantum algorithms that fold on stochastic multidimensional financial models. Each type of calculation requires a new algorithm to be written. Unlike traditional computers, quantum computers require specially written algorithms to perform tasks in their unique environment. The key challenge for quantum computers is to create algorithms that can effectively solve complex issues. Quantum computer algorithms are frequently compared to classical computing logic.
3. *Taste and complete the algorithms*—The process involves both testing and refining the algorithm, as well as its implementation. Additionally, the performance of the quantum algorithm is compared to classical algorithms to evaluate its potential advantages. During this phase, classical and quantum algorithms are compiled simultaneously, following the model in which quantum computing can be utilized to enhance existing financial systems and practices.
4. *Launch and improve the simulation*—The implementation process concludes with the release of the solution. To handle larger and more complex financial data and tasks, the quantum algorithm and its implementation need to be scaled up. Furthermore, there is a need for ongoing refinement and optimization of the algorithm to enhance its effectiveness and efficiency.

### (c) Quantum Finance Use Cases

Quantum finance involves using mathematical and statistical models to identify patterns and trends in market data, as well as to build predictive models that can help anticipate future price movements. It also involves using technology to process data in real time so that investors can make quick and accurate investment decisions. Some examples of mathematical and statistical methods used in the field of quantum finance include:

- *Statistical analysis*—involves using statistical methods to analyze historical market data and identify trends and patterns.
- *Time series analysis*—involves analyzing historical price and volume data and building mathematical models that can be used to predict future price movements.
- *Factor analysis*—involves identifying factors that may influence market prices and building mathematical models that can be used to predict future price movements.
- *Neural network analysis*—involves using mathematical models inspired by the structure of the human brain to analyze market data and make predictions.
- *Portfolio theory*—involves using mathematical models to construct investment portfolios that maximize returns and minimize risk.
- *Spectral analysis*—involves analyzing market data by frequency and using this information to make predictions.
- *Fractal analysis*—involves analyzing market data using fractal geometry and building mathematical models that can be used to make predictions.

Quantum finance is commonly used by financial institutions which seek to gain a competitive advantage in making investment decisions. Capital markets underline using quantum finance for portfolio optimization, risk assessment, options pricing, financial fraud detection, high-frequency trading, and quantum cryptography. Banks aim to implement various steps such as analyzing their risk profile, scoring loans, and improving investment strategies with the help of quantum computing. The efforts of major banking giants including Barclays, HSBC, Goldman Sachs, and JPMorgan Chase to implement quantum computing are being bolstered by technology giants such as Intel, Google, and IBM, who are announcing advancements in the number of available qubits in their quantum computing architectures. JPMorgan Chase, for instance, has shown that a 20-qubit quantum computer can effectively utilize natural language processing (NLP) to summarize large numbers of documents. Traders must pore over many documents every morning to make the most informed investment decisions, and this technology could help them do so more efficiently. The capital market is often referred to as the Queen of Finance due to its high volatility resulting from the massive influx of information that impacts investors’ decisions. For instance, JPMorgan Chase has showcased the practicality of its NISQ-HHL quantum algorithm for optimizing investment portfolios.

The prospect of the future of quantum computing only increases the interest in quantum finance and numerous proposed applications in fields such as quantum chemistry, pharmacy, healthcare, artificial intelligence, security, logistics, aeronautics, and industry.

## 6. Conclusions

The language of quantum computing in finance is a novel line of progress that is emerging that integrates existing multidimensional financial data with non-existing information and blends it with advancements in Artificial Intelligence, Big Data, and Machine Learning to generate precise financial predictions. Computers are on the verge of playing a pivotal role in shaping the progress of emerging technologies soon, more robust, and efficient technologies. With quantum computing, you can have a bird’s-eye view and observe all possible paths at the same time. While the theory behind quantum finance is sound, experts agree that it is still a field that requires time, experimentation, and refinement in practice. According to a press release from Forbes, multiple players are developing their quantum models, but no definitive solution has emerged yet.

By summarizing the status of the field in a thorough framework that highlights research problems already addressed, our study adds to the body of knowledge on quantum finance. Our literature review may offer further research issues that could direct investigations into the application of quantum computing in contemporary finance. Additional work is required to define the concrete contribution of quantum theory for financial institutions and individuals interested in financial markets. Quantum finance research is open to new ideas of theoretical and empirical modeling. To sum up, quantum computing has the potential to revolutionize the finance industry by enabling faster and more accurate analysis of vast amounts of financial data. It can enhance risk analysis, fraud detection, and algorithmic trading, and lead to the development of new security methods and optimization techniques. With major banking giants and technology companies investing in the development of quantum computing, the future of finance is tightly intertwined with the future of quantum computing. As quantum computing continues to evolve and become more accessible, we can expect to see even more innovative applications in finance and other industries.

In conclusion, quantum finance has the potential to address some of the limitations of traditional finance methods by using quantum algorithms that can process more complex data and consider multiple outcomes simultaneously. It is an emerging field that is still being explored, and more research is needed to fully understand its potential applications and limitations.

Like any research paper, our systematic literature review has several limitations related to the selection process of articles and the subjectivity of the content evaluation. Among the limitations, it can be found the following: the review is limited to papers indexed in only two databases, the review does not include grey literature and books, the review does nu consider literature in other languages than English, or full-text papers are not available because of open-access restrictions. Another constraint is represented by the sampling errors that may appear in a systematic literature review. For instance, some studies are unintentionally omitted because authors apply quantum algorithms to study financial areas, but the papers do not contain ‘quantum finance’ in the query fields. In this case, they are not found during the current search strategy. In this research, we have tried to select those papers that have quantum finance in the foreground and any other financial areas in the background. In addition to the constraints mentioned above, it should be mentioned that the findings of the research should be interpreted considering the limited number of studies included in the sample and the appropriate level of representativeness. All these limitations can be addressed in future literature reviews on quantum finance. Regarding our future research perspective, the current study may be improved and developed by a bibliometric analysis and by using visualization software to map the knowledge in quantum finance. Studying co-authorship, co-citation, and keywords co-occurrence could lead to additional and precious information in quantum finance. On the other hand, the analysis can be extended by widening the sample and modifying the selection strategy to include articles that have a relevant connection with quantum finance. Finally, another aspect that can be the subject of future research is a deepening of the theories about quantum finance that already exist in the literature.

## Supporting information

S1 Checklist PRISMA checklist. (DOCX)

S1 Appendix Papers selected in the systematic literature review. (DOCX)

S2 Appendix Synthetic exposure of quantum finance theories according to previous research. (DOCX)
