# Supplementary material for: Modern finance through quantum computing—A systematic literature review
Source: PLoS One. 2024 Jul 18;19(7):e0304317. doi: 10.1371/journal.pone.0304317 (PMC11257328; doi:10.1371/journal.pone.0304317)
Supplement: S1 Appendix — (DOCX) [file pone.0304317.s002.docx]

**S1 Appendix. Papers selected in the systematic literature review**

| **No** | **Authors** | **Title** | **Journal** | **DOI** |
| --- | --- | --- | --- | --- |
| 1 | Aboussalah, AM; Chi, C; Lee, CG (2023) | Quantum computing reduces systemic risk in financial networks | Scientific Reports | https://doi.org/10.1038/s41598-023-30710-z |
| 2 | Alcazar, J; Leyton-Ortega, V; Perdomo-Ortiz, A (2020) | Classical versus quantum models in machine learning: insights from a finance application | Machine Learning: Science and Technology | 10.1088/2632-2153/ab9009 |
| 3 | An, D; Linden, N; Liu, JP; Montanaro, A; Shao, CP; Wang, JS (2021) | Quantum-accelerated multilevel Monte Carlo methods for stochastic differential equations in mathematical finance | Quantum | https://doi.org/10.48550/arXiv.2012.06283 |
| 4 | Araújo R.D.A., De Oliveira A.L.I., Soares S.C.B. (2010a) | Hybrid evolutionary quantum inspired method to adjust time phase distortions in financial time series | Conference -Proceedings of the 2010 ACM Symposium on Applied Computing | https://doi.org/10.1145/1774088.1774332 |
| 5 | Araujo, RDA; de Oliveira, ALI; Soares, SCB (2010b) | A Quantum-Inspired Hybrid Methodology for Financial Time Series Prediction | Conference - International Joint Conference on Neural Networks | [10.1109/IJCNN.2010.5604601](http://dx.doi.org/10.1109/IJCNN.2010.5604601) |
| 6 | Arraut, I; Au, A; Tse, ACB (2020) | Spontaneous symmetry breaking in quantum finance | A Letters Journal Exploring The Frontiers of Physics | <https://doi.org/10.48550/arXiv.2011.05278> |
| 7 | Arraut, I; Au, A; Tse, ACB; Segovia, C (2019) | The connection between multiple prices of an Option at a given time with single prices defined at different times: The concept of weak-value in quantum finance | Physica A | <https://doi.org/10.1016/j.physa.2019.04.264> |
| 8 | Arraut, I; Marques, JAL; Gomes, S (2021) | The Probability Flow in the Stock Market and Spontaneous Symmetry Breaking in Quantum Finance | Mathematics | <https://doi.org/10.3390/math9212777> |
| 9 | Baaquie B.E. (2008a) | Quantum finance Hamiltonian for coupon bond European and barrier options | Physical Review E | [10.1103/PhysRevE.77.036106](https://doi.org/10.1103/physreve.77.036106) |
| 10 | Baaquie, BE (2018) | Bonds with index-linked stochastic coupons in quantum finance | Physica A | <https://doi.org/10.1016/j.physa.2018.02.003> |
| 11 | Baaquie, BE (2007) | Feynman perturbation expansion for the price of coupon bond options and swaptions in quantum finance. I. Theory | Physical Review E | [10.1103/PhysRevE.75.016703](http://dx.doi.org/10.1103/PhysRevE.75.016703) |
| 12 | Baaquie, BE (2013) | Financial modeling and quantum mathematics | Computers and Mathematics with Applications | <https://doi.org/10.1016/j.camwa.2013.01.025> |
| 13 | Baaquie, BE (2010) | Interest rates in quantum finance: Caps, swaptions and bond options | Physica A | <https://doi.org/10.1016/j.physa.2009.09.031> |
| 14 | Baaquie, BE (2009) | Interest rates in quantum finance: The Wilson expansion and Hamiltonian | Physical Review E | [10.1103/PhysRevE.80.046119](http://dx.doi.org/10.1103/PhysRevE.80.046119) |
| 15 | Baaquie, BE (2008b) | Quantum finance Hamiltonian for coupon bond European and barrier options | Physical Review E | [10.1103/PhysRevE.77.036106](http://dx.doi.org/10.1103/PhysRevE.77.036106) |
| 16 | Baaquie, BE; Du, X; Tang, P; Cao, Y (2014) | Pricing of range accrual swap in the quantum finance Libor Market Model | Physica A | <https://doi.org/10.1016/j.physa.2014.01.042> |
| 17 | Baaquie, BE; Liang, C (2007a) | Feynman perturbation expansion for the price of coupon bond options and swaptions in quantum finance. II. Empirical | Physical Review E | [10.1103/PhysRevE.75.016703](http://dx.doi.org/10.1103/PhysRevE.75.016703) |
| 18 | Baaquie, BE; Liang, C (2007b) | Pricing American options for interest rate caps and coupon bonds in quantum finance | Physica A | <https://doi.org/10.1016/j.physa.2007.02.054> |
| 19 | Baaquie, BE; Pan, T (2011) | Simulation of coupon bond European and barrier options in quantum finance | Physica A | <https://doi.org/10.1016/j.physa.2010.08.046> |
| 20 | Baaquie, BE; Tang, P (2012) | Simulation of nonlinear interest rates in quantum finance: Libor Market Model | Physica A | <https://doi.org/10.1016/j.physa.2011.08.021> |
| 21 | Baaquie, BE; Yang, C (2009) | Empirical analysis of quantum finance interest rates models | Physica A | <https://doi.org/10.1016/j.physa.2009.02.044> |
| 22 | Baaquie, BE; Yu, M; Bhanap, J (2018) | Risky forward interest rates and swaptions: Quantum finance model and empirical results | Physica A | <https://doi.org/10.1016/j.physa.2017.09.045> |
| 23 | Bagheri, A; Peyhani, HM; Akbari, M (2014) | Financial forecasting using ANFIS networks with Quantum-behaved Particle Swarm Optimization | Expert Systems with Applications | <https://doi.org/10.1016/j.eswa.2014.04.003> |
| 24 | Bai, L; Cui, LX; Wang, Y; Jiao, YH; Hancock, ER (2020) | A Quantum-inspired Entropic Kernel for Multiple Financial Time Series Analysis | Conference - Proceedings of the Twenty-Ninth International Joint Conference on Artificial Intelligence (IJCAI-20) | <https://doi.org/10.24963/ijcai.2020/614> |
| 25 | Barad, G (2012) | Classical and quantum symmetries in option pricing; a theoretical approach to risk and randomness in finance | Conference - International Conference IMAR, 2011, Bucharest | http://www.imar.ro/~purice/Inst/2011/CERBUN-1/GBarad.pdf |
| 26 | Biesner, D; Gerlach, T; Sifa, R; Bauckhage, C; Kliem, B (2022) | Solving Subset Sum Problems using Quantum Inspired Optimization Algorithms with Applications in Auditing and Financial Data Analysis | Conference - proceedings of IEEE International Conference on Machine Learning Applications IEEE ICMLA 2022 | https://doi.org/10.48550/arXiv.2211.02653 |
| 27 | Chang B.R., Tsai H.F. (2006a) | New approach to financial time series forecasting - Quantum minimization regularizing BWGC and NGARCH composite model | Conference - Proceedings of the 9th Joint International Conference on Information Sciences (JCIS-06) | [10.2991/jcis.2006.125](https://doi.org/10.2991/jcis.2006.125) |
| 28 | Chang, BR; Tsai, HF (2006b) | Financial prediction applications using quantum-minimized composite model ASVR/NGARCH | Conference - 2006 International Joint Conference on Neural Networks | [10.1109/IJCNN.2006.246834](http://dx.doi.org/10.1109/IJCNN.2006.246834) |
| 29 | Chang, BR; Tsai, HF (2009) | Quantum-minimized BWGC/NGARCH approach to financial time series forecast | Neurocomputing | <https://doi.org/10.1016/j.neucom.2008.11.002> |
| 30 | Chen, CM; Tso, GKF; He, KJ (2023) | Quantum Optimized Cost Based Feature Selection and Credit Scoring for Mobile Micro-financing | Computational Economics | https://doi.org/10.1007/s10614-023-10365-8 |
| 31 | Choustova, O. (2007a) | Quantum-like models in economics and finances | Conference - American Association for Artificial Intelligence - 2007 Symposion | https://cdn.aaai.org/Symposia/Spring/2007/SS-07-08/SS07-08-022.pdf |
| 32 | Choustova, O. (2007b) | Toward quantum-like modeling of financial processes | Journal of Physics | 10.1088/1742-6596/70/1/012006 |
| 33 | Choustova, O (2009) | Quantum probability and financial market | Information Sciences | <https://doi.org/10.1016/j.ins.2008.07.001> |
| 34 | Choustova, O (2007c) | Quantum Bohmian model for financial market | Physica A | <https://doi.org/10.1016/j.physa.2006.07.029> |
| 35 | Covers O., Doeland M. | How the financial sector can anticipate the threats of quantum computing to keep payments safe and secure | Journal of Payments Strategy & Systems | https://www.betaalvereniging.nl/wp-content/uploads/Quantum-computing-keep-payments-safe-and-secure.pdf |
| 36 | Coyle, B; Henderson, M; Le, JCJ; Kumar, N; Paini, M; Kashefi, E (2020) | Quantum versus classical generative modelling in finance | Quantum Science and Technology | <https://doi.org/10.48550/arXiv.2008.00691> |
| 37 | Cruz, P; Cruz, H (2020) | Piecewise Linear Representation of Finance Time Series: Quantum Mechanical Tool | Acta Physica Polonica A | : 10.12693/APhysPolA.138.21 |
| 38 | Darbyshire, P (2005) | Quantum physics meets classical finance | Physics World | [10.1088/2058-7058/18/5/36](http://dx.doi.org/10.1088/2058-7058/18/5/36) |
| 39 | Ding, YC; Gonzalez-Conde, J; Lamata, L; Martin-Guerrero, JD; Lizaso, E; Mugel, S; Chen, X; Orus, R; Solano, E; Sanz, M (2023) | Toward Prediction of Financial Crashes with a D-Wave Quantum Annealer | Entropy | <https://doi.org/10.3390/e25020323> |
| 40 | Doriguello J.F., Luongo A., Bao J., Rebentrost P., Santha M. (2022) | Quantum Algorithm for Stochastic Optimal Stopping Problems with Applications in Finance | Conference - 17th Conference on the Theory of Quantum Computation, Communication and Cryptography (TQC 2022) | <https://doi.org/10.4230/LIPIcs.TQC.2022.2> |
| 41 | Dupoyet, B; Fiebig, HR; Musgrove, DP (2010) | Gauge invariant lattice quantum field theory: Implications for statistical properties of high frequency financial markets | Physica A | <https://doi.org/10.1016/j.physa.2009.09.002> |
| 42 | Farinelli S., Takada H. (2022) | When risks and uncertainties collide: Quantum mechanical formulation of mathematical finance for arbitrage markets | Conference Proceedings | http://dx.doi.org/10.2139/ssrn.3404437 |
| 43 | Feng, XN; Wu, HY; Zhou, XL; Yao, Y (2022) | Quantum blind signature scheme for supply chain financial | Quantum Information Processing | https://doi.org/10.1007/s11128-022-03763-8 |
| 44 | Fernandez-Lorenzo, S; Porras, D; Garcia-Ripoll, JJ (2021) | Hybrid quantum-classical optimization with cardinality constraints and applications to finance | Quantum Science and Technology | <https://doi.org/10.48550/arXiv.2008.12050> |
| 45 | Fontanela, F; Jacquier, A; Oumgari, M (2019) | Short Communication: A Quantum Algorithm for Linear PDEs Arising in Finance | SIAM Journal on Financial Mathematics | <https://doi.org/10.48550/arXiv.1912.02753> |
| 46 | Ghosh B., Kozarevic E. (2018) | Identifying explosive behavioral trace in the CNX nifty index: A quantum finance approach | Investment Management and Financial Innovations | https://papers.ssrn.com/sol3/papers.cfm?abstract_id=3135035 |
| 47 | Gomez, A; Leitao, A; Manzano, A; Musso, D; Nogueiras, MR; Ordonez, G; Vazquez, C (2022) | A Survey on Quantum Computational Finance for Derivatives Pricing and VaR | Archives of Computational Methods in Engineering | https://doi.org/10.1007/s11831-022-09732-9 |
| 48 | Goncalves, CP (2011) | Quantum financial economics - risk and returns | Quantum | <https://doi.org/10.48550/arXiv.1107.2562> |
| 49 | Griffin, P; Sampat, R (2021) | Quantum Computing for Supply Chain Finance | Conference - 2021 IEEE International Conference on Services Computing (SCC) | doi: 10.1109/SCC53864.2021.00066 |
| 50 | Hanauske, M; Kunz, J; Bernius, S; Konig, W (2010) | Doves and hawks in economics revisited: An evolutionary quantum game theory based analysis of financial crises | Physica A | <https://doi.org/10.1016/j.physa.2010.06.007> |
| 51 | Haven E. (2007) | A survey of possible uses of quantum mechanical concepts in financial economics | Quantum | https://arxiv.org/pdf/2201.02773.pdf |
| 52 | Haven E. (2019) | Finance and the quantum mechanical formalism | Studies in Computational Intelligence | [10.1007/978-3-030-04200-4_4](http://dx.doi.org/10.1007/978-3-030-04200-4_4) |
| 53 | Hellstem, G (2021) | Hybrid Quantum Network for classification of finance and MNIST data | Conference - 2021 IEEE 18th International Conference on Software Architecture Companion (ICSA-C) | <https://ieeexplore.ieee.org/document/9425825> |
| 54 | Henkel, C (2017) | From quantum mechanics to finance: Microfoundations for jumps, spikes and high volatility phases in diffusion price processes | Physica A | <https://doi.org/10.1016/j.physa.2016.11.125> |
| 55 | Hwang J.H. (2015) | Risk quanta: an approach to understanding modern financial risk | Journal of Financial Regulation and Compliance | <https://doi.org/10.1108/JFRC-02-2014-0015> |
| 56 | Ingber L. (2015) | Hybrid classical-quantum computing: Applications to statistical mechanics of financial markets | Conference - E3S Web of Conferences | <http://dx.doi.org/10.2139/ssrn.3828630> |
| 57 | Kaneko, K; Miyamoto, K; Takeda, N; Yoshino, K (2021) | Quantum speedup of Monte Carlo integration with respect to the number of dimensions and its application to finance | Quantum Information Processing | https://doi.org/10.1007/s11128-021-03127-8 |
| 58 | Khrennikov, A (2007) | Classical and Quantum-Like Randomness and the Financial Market | Quantum | <https://doi.org/10.48550/arXiv.0704.2865> |
| 59 | Khrennikova P. (2019) | Quantum-like model of subjective expected utility: A survey of applications to finance | Beyond Traditional Probabilistic Methods in Economics | [10.1007/978-3-030-04200-4_5](http://dx.doi.org/10.1007/978-3-030-04200-4_5) |
| 60 | Kim, MJ; Hwang, DI; Lee, SY; Kim, SY (2011) | The sensitivity analysis of propagator for path independent quantum finance model | Physica A | <https://doi.org/10.1016/j.physa.2010.11.016> |
| 61 | Mancilla, J; Pere, C (2022) | A Preprocessing Perspective for Quantum Machine Learning Classification Advantage in Finance Using NISQ Algorithms | Entropy | <https://doi.org/10.3390/e24111656> |
| 62 | Manjunath C., Marimuthu B., Ghosh B. (2023) | Analysis of Nifty 50 index stock market trends using hybrid machine learning model in quantum finance | International Journal of Electrical and Computer Engineering (IJECE) | <http://doi.org/10.11591/ijece.v13i3.pp3549-3560> |
| 63 | Martin, A; Candelas, B; Rodriguez-Rozas, A; Martin-Guerrero, JD; Chen, X; Lamata, L; Orus, R; Solano, E; Sanz, M (2019) | Toward pricing financial derivatives with an IBM quantum computer | Physical Review Research | <https://doi.org/10.48550/arXiv.1904.05803> |
| 64 | Melnyk S.I., Tuluzov I.G. (2008) | Quantum analog of the Black-Scholes formula (market of financial derivatives as a continuous weak measurement) | Electronic Journal of Theoretical Physics | https://philarchive.org/archive/MELQAO |
| 65 | Mugel S., Lizaso E., Orús R. (2020) | Use Cases of Quantum Optimization for Finance | Quantum Computing in Econometrics and Quantum Economics and Related Topics Quantum Computing in Econometrics and Quantum Economics and Related Topics | <https://doi.org/10.48550/arXiv.2010.01312> |
| 66 | Nakaji, K; Uno, S; Suzuki, Y; Raymond, R; Onodera, T; Tanaka, T; Tezuka, H; Mitsuda, N; Yamamoto, N (2021) | Approximate amplitude encoding in shallow parameterized quantum circuits and its application to financial market indicators | Physical Review Research | <https://doi.org/10.1103/PhysRevResearch.4.023136> |
| 67 | Nakayama, Y (2009) | GRAVITY DUAL FOR REGGEON FIELD THEORY AND NONLINEAR QUANTUM FINANCE | International Journal of Modern Physics A | <https://doi.org/10.48550/arXiv.0906.4112> |
| 68 | Nastasiuk, VA (2014) | Emergent quantum mechanics of finances | Physica A | <https://doi.org/10.1016/j.physa.2014.02.037> |
| 69 | Nastasiuk, VA (2015) | Fisher information and quantum potential well model for finance | Physics Letters A | <https://doi.org/10.1016/j.physleta.2015.06.052> |
| 70 | Orús R., Mugel S., Lizaso E. (2019) | Quantum computing for finance: Overview and prospects | [Reviews in Physics](https://www.sciencedirect.com/journal/reviews-in-physics) | <https://doi.org/10.1016/j.revip.2019.100028> |
| 71 | Orus, R; Mugel, S; Lizaso, E (2018) | Forecasting financial crashes with quantum computing | Physical Review A | <https://doi.org/10.48550/arXiv.1810.07690> |
| 72 | Pan, WT; Liu, Y; Jiang, H; Chen, YT; Liu, T; Qing, Y; Huang, GH; Li, R (2021) | Model Construction of Enterprise Financial Early Warning Based on Quantum FOA-SVR | Scientific Programming | [10.1155/2021/5018917](http://dx.doi.org/10.1155/2021/5018917) |
| 73 | Paquet, E; Soleymani, F (2022) | QuantumLeap: Hybrid quantum neural network for financial predictions | Expert Systems with Applications | <https://doi.org/10.1016/j.eswa.2022.116583> |
| 74 | Petrenko, K; Mashatan, A; Shirazi, F (2019) | Assessing the quantum-resistant cryptographic agility of routing and switching IT network infrastructure in a large-size financial organization | Journal of Information Security and Applications | <https://doi.org/10.1016/j.jisa.2019.03.007> |
| 75 | Piotrowski, EW; Sladkowski, J (2004) | Quantum games in finance | Quantitative Finance | [10.1080/14697680400014344](http://dx.doi.org/10.1080/14697680400014344) |
| 76 | Piotrowski, EW; Sladkowski, J (2001) | Quantum-like approach to financial risk: Quantum anthropic principle | Acta Physica Polonica B | <https://doi.org/10.48550/arXiv.quant-ph/0110046> |
| 77 | Pistoia, M; Ahmad, SF; Ajagekar, A; Buts, A; Chakrabarti, S; Herman, D; Hu, SH; Jena, A; Minssen, P; Niroula, P; Rattew, A; Sun, Y; Yalovetzky, R (2021) | Quantum Machine Learning for Finance ICCAD Special Session Paper | Quantum | <https://doi.org/10.48550/arXiv.2109.04298> |
| 78 | Qiu Y., Liu R., Lee R.S.T. (2021) | The design and implementation of quantum finance-based hybrid deep reinforcement learning portfolio investment system | Journal of Physics | <https://iopscience.iop.org/article/10.1088/1742-6596/1828/1/012011> |
| 79 | Racorean O. (2013) | Are financial markets an aspect of quantum world? | Quantum | <https://doi.org/10.48550/arXiv.1305.1559> |
| 80 | Rebentrost, P; Gupt, B; Bromley, TR (2018) | Quantum computational finance: Monte Carlo pricing of financial derivatives | Physical Review A | [10.1103/PhysRevA.98.022321](http://dx.doi.org/10.1103/PhysRevA.98.022321) |
| 81 | Romero J.M., Lavana U., Miranda E.M. (2014) | Schrödinger group and quantum finance | International Journal of Pure and Applied Mathematics | [10.12732/ijpam.v90i3.3](http://dx.doi.org/10.12732/ijpam.v90i3.3) |
| 82 | Romero, JM; Miranda, EM; Lavana, U (2014) | Conformal symmetry in quantum finance | Journal of Physics | [10.1088/1742-6596/512/1/012029](http://dx.doi.org/10.1088/1742-6596/512/1/012029) |
| 83 | Sarkissian, J (2020) | Quantum coupled-wave theory of price formation in financial markets: Price measurement, dynamics and ergodicity | Physica A | <https://doi.org/10.1016/j.physa.2020.124300> |
| 84 | Schaden, M (2002) | Quantum finance | Physica A | <https://doi.org/10.48550/arXiv.physics/0203006> |
| 85 | Singh, S; Subrahmanya, MHB (2021) | Quantum of finance obtained by tech startups over the lifecycle: an analysis of its determinants | International Review of Applied Economics | <https://doi.org/10.1080/02692171.2021.1945549> |
| 86 | Stamatopoulos, N; Mazzola, G; Woerner, S; Zeng, WJ (2021) | Towards Quantum Advantage in Financial Market Risk using Quantum Gradient Algorithms | Quantum | <https://doi.org/10.48550/arXiv.2111.12509> |
| 87 | Sun, J; Xu, WB; Fang, W (2010) | Solving multi-period financial planning problem via quantum-behaved particle swarm algorithm | Computational Intelligence | https://doi.org/10.1007/978-3-540-37275-2_143 |
| 88 | Tahmasebi, F; Meskinimood, S; Namaki, A; Farahani, SV; Jalalzadeh, S; Jafari, GR (2015) | Financial market images: A practical approach owing to the secret quantum potential | A Letters Journal Exploring The Frontiers of Physics | [10.1209/0295-5075/109/30001](http://dx.doi.org/10.1209/0295-5075/109/30001) |
| 89 | Tang, YH; Yan, JC; Hu, GQ; Zhang, BH; Zhou, JZ (2022) | Recent progress and perspectives on quantum computing for finance | Service Oriented Computing and Applications | https://doi.org/10.1007/s11761-022-00351-7 |
| 90 | Wang, CF; Yang, YK; Xu, LL; Wong, A (2023) | A Hybrid Model of Primary Ensemble Empirical Mode Decomposition and Quantum Neural Network in Financial Time Series Prediction | Fluctuation and Noise Letters | <https://doi.org/10.1142/S0219477523400060> |
| 91 | Wilkens, S; Moorhouse, J (2023) | Quantum computing for financial risk measurement | Quantum Information Processing | https://doi.org/10.1007/s11128-022-03777-2 |
| 92 | Yaghobipour, S; Yarahmadi, M (2018) | Optimal control design for a class of quantum stochastic systems with financial applications | Physica A | <https://doi.org/10.1016/j.physa.2018.08.141> |
| 93 | Yaghobipour, S; Yarahmadi, M (2020) | Solving quantum stochastic LQR optimal control problem in Fock space and its application in finance | Computers and Mathematics with Applications | <https://doi.org/10.1016/j.camwa.2019.12.016> |
| 94 | Yeşiltaş Ö. (2023) | The Black–Scholes equation in finance: Quantum mechanical approaches | Physica A | <https://doi.org/10.1016/j.physa.2023.128909> |
