# Supplementary material for: Modern finance through quantum computing—A systematic literature review
Source: PLoS One. 2024 Jul 18;19(7):e0304317. doi: 10.1371/journal.pone.0304317 (PMC11257328; doi:10.1371/journal.pone.0304317)
Supplement: S2 Appendix — (DOCX) [file pone.0304317.s003.docx]

**S2 Appendix. Synthetic exposure of quantum finance theories according to previous research**

| **ID paper** | **Quantum finance model category** | **What theory of quantum finance is targeted by the model?** | **What are the particularities of the quantum model?** | **What problems does the quantum model solve?**  **What are the limits of the quantum model?**  **What are the future challenges?** |
| --- | --- | --- | --- | --- |
| **ID1** | Risk Management | The proposed model manages systemic risk, partitioning problems, and network optimization in the interbank environment. | It is a two-stage optimization algorithm, that compares classical and quantum algorithms and integrates global and local risk. | It is more resilient to financial shocks, delays the cascade failure phase transition, and reduces the total number of failures at convergence under systemic risks with reduced time complexity. |
| **ID2** | Modeling Techniques | Comparison of the classical ML models known as restricted Boltzmann machines (RBMs), against a quantum model, known as quantum circuit Born machines (QCBMs). | The quantum learning pipeline; the classical learning approach; analogy with statistical mechanics, comparative performance; | The quantum models seem to have superior performance on typical instances when compared with the canonical training of the RBMs. |
| **ID3** | Financial Algorithms | Quantum algorithms for stochastic differential equations (SDEs) and binomial option pricing model. | Provide a quantum algorithm that gives a quadratic speed-up for multilevel Monte Carlo methods in a general setting, a quantum algorithm based on sublinear binomial sampling for the binomial option pricing model; Quantum-accelerated MLMC is used for solving SDEs; A standard multilevel Monte Carlo method was applied | Demonstrates the use of the algorithm in a variety of applications, such as the Black-Scholes and Local Volatility models, and Greeks. It is limited to parabolic PDEs as an application for simulating SDEs. Future challenges are the application of the algorithm to Poisson’s equation, elliptic PDEs, or more general PDEs, to provide other meaningful characteristics of stochastic processes, and to find more practical quantum input-output models for potential applications in finance. |
| **ID4** | Prediction Techniques | A hybrid quantum-inspired financial time series analysis to adjust time phase distortions, overcoming the random walk dilemma for financial predictions | The model composes a Qubit Multilayer Perceptron (QuMLP) with a Quantum Inspired Evolutionary Algorithm (QIEA), it searches for the best time lags to describe the time series generator phenomenon. | It demonstrates a better performance when compared to the MLP networks, and better performance than the TAEF method model. The model was able to adjust the time phase delay, the MLP models were not capable of producing such correction behavior although the same procedure was applied to all the models. Future challenge - to better formalize and explain the properties of the proposed model and to use it with other financial time series, including components such as trends, seasonality, impulses, steps, and other nonlinearities. |
| **ID5** | Prediction Techniques | A quantum-inspired hybrid methodology to overcome the random walk dilemma for financial time series prediction | A hybrid model composed of a Qubit Multilayer Perceptron (QuMLP) with a Quantum-Inspired Evolutionary Algorithm (QIEA), which searches for the best time lags able to characterize the time series phenomenon, as well as to evolve the complete QuMLP architecture and parameters. | The model obtained a much better performance than a previous random walk model for the analyzed financial time series. The model can adjust the time-phase delay, the MLP models were not capable of producing such correction behavior although the same procedure was applied. |
| **ID6** | Theory Applications | The phenomena of spontaneous symmetry breaking in Quantum Finance by using as a starting point the Black-Scholes (BS) and the Merton-Garman (MG) equations expressed in the Hamiltonian form. | The Martingale condition is a vacuum state that becomes degenerate when the symmetry of the system is spontaneously broken. Martingale's condition is extended to include the stochastic volatility for the Merton Garman case. | It demonstrates that another way to analyze these equations is by considering the concept of spontaneous symmetry breaking. When both equations are expressed as a Hamiltonian equation of the Schrödinger type, then it comes out that the martingale state is the ground (vacuum) state of the system. Instead of identifying two events affecting the Stock market as different, they can be connected by using the concept of spontaneous symmetry breaking proposed. The model can be used to some predictions in the market. |
| **ID7** | Prediction Techniques | Prediction of the evolution of an option for the cases where at some specific time, there is a high degree of uncertainty in identifying its price; Weak-value approach in quantum finance | The model explains the standard double-slit experiment and its weak-value representation. It introduces comments on non-Hermitian Hamiltonians and their natural appearance in open systems, weak value for operators evolving in agreement with non-Hermitian Hamiltonians. The concept of weak value is applied to the Black–Scholes Hamiltonian. | It introduces the possibility of modeling the uncertainties in the prices of the options by using the double-slit approach and how to use the concept of weak value for this purpose. The concept of weak value certainly simplifies the analysis of the evolution of the prices, and it will be a powerful tool for applications connected with Machine (and Deep) learning. |
| **ID8** | Theory Applications | The spontaneous symmetry-breaking phenomena under changes in prices and volatility | The model analyzes the effective symmetry-breaking patterns and the connected vacuum degeneracy, the connection between the flow of information and the multiplicity of martingale states, providing tools for analyzing the dynamic of the stock markets. | A deeper analysis of the Black–Scholes and the Merton–Garman Equation was not considered before in previous papers. The use of symmetry arguments to analyze the flow of information in the stock market and equilibrium conditions proposed can be complemented, with the formalism employing fractional functionals in order to have a deeper visualization of the flow of information in the market. |
| **ID9** | Modeling Techniques | The European and double barrier options are defined and reexpressed in a form suitable for an approximate computation of their price. | The Hamiltonian, state space, and the evolution operator for the forward interest rates are discussed. European coupon bond option price is rederived using the Hamiltonian formulation. The price of the barrier option is defined using the concept of state space and matrix elements of the evolution operator. A consistent linearization of the coupon bond payoff function is discussed and an overcomplete set of eigenfunctions, necessary for computing the approximate coupon bond barrier option price is introduced. | The Hamiltonian formulation of the quantum field theory of forward interest rates provides an efficient computation tool for analyzing the coupon bond European and barrier options. |
| **ID10** | Theory Applications | Bonds with index-linked stochastic coupons in quantum finance | Stochastic coupon’s payoff function, Hamiltonian for the index, Feynman path integral and pricing kernel, price of the stochastic coupon, Martingale condition, put-call parity | The model can be generalized to include stochastic volatility by using the Merton–Garman Hamiltonian. It can be further extended to the case where not only are the amount of the coupon payments, but the payments of the principal as well as the tenor of the coupon bond are also stochastic. |
| **ID11** | Modeling Techniques | Approximation of coupon bond option price since the volatility of the forward interest rates is a small quantity. | The coupon bond option price in the one-factor HJM model with exponential volatility is compared with the HJM limit of the approximate field theory coupon bond option price. A special case of the approximate bond option is shown to yield the industry standard one-factor HJM formula with exponential volatility. The perturbation expansion using Feynman diagrams is realized by expanding the nonlinear terms in the partition function and performing the path integral order by order using Gaussian path integrations. | A perturbation expansion using Feynman diagrams gives a closed-form approximation for the price of the coupon bond option. The approximate coupon bond option price shows that the correlation between the forward prices of bonds of different maturities plays a crucial role in yielding an accurate price for the swaptions. |
| **ID12** | Modeling Techniques | Modeling financial instruments with quantum mechanics | Financial instruments are described by the elements of a linear vector state space and its evolution is determined by a Hamiltonian operator. Forward interest rates empirically observed imperfect and nontrivial correlations are accurately modeled by quantum field theory. | It shows that interest rates can be described by a random function – which is mathematically equivalent to a two-dimensional Euclidean quantum field. Future challenge – to demonstrate the usefulness of quantum finance models for practitioners of finance. |
| **ID13** | Modeling Techniques | Modelling interest rate options in the financial markets, derived from the Libor (London Interbank Overnight Rate), by using a quantum finance model | Libor Market Model coupon bond option price, Libor swaption and bond option model, European swaption price, Libor Asian swaption price, BGM-Jamshidian swaption price, Black’s caplet formula. The approximate Libor option prices are derived using the volatility expansion. The caplet price was exactly evaluated and provides a quantum finance generalization of Black’s formula. | Swaption price is a nonlinear problem for the quantum finance Libor Market Model. The Libor European and Asian swaption prices were obtained as a perturbation expansion in the log Libor volatility and demonstrates the crucial role of the Libor correlator in pricing these instruments. Two different approximate Libor Market Model swaption prices have been obtained, but they need to be empirically studied to decide which is best suited for applications. |
| **ID14** | Modeling Techniques | The quantum finance formulation of the Libor market model | The Lagrangian and Feynman path integrals of the Libor market model of interest rates were obtained, as well as a derivation given by its Hamiltonian. The Hamiltonian formulation of the martingale condition provided an exact solution for the nonlinear drift of the Libor market model. The Hamiltonian is the appropriate framework for imposing the martingale condition for nonlinear interest rates and yields the exact expression for the Libor market model’s nonlinear drift. | All the Libors, for different future times, are imperfectly correlated. A key difference between a forward interest rate model and the LMM lies in the fact that the LMM is calibrated directly from the observed market interest rates. The quantum finance formulation of the LMM is shown to reduce to the industry standard Bruce-Gatarek-Musiela-Jamshidian model when the forward interest rates are taken to be exactly correlated. A new feature of the interest rate dynamics is that the interest rates’ state space and Hamiltonian are time dependent. |
| **ID15** | Modeling Techniques | Analysis of coupon bond European and barrier options in the Hamiltonian formulation of quantum finance. | Forward interest rates are modeled as a two-dimensional quantum field theory and its Hamiltonian and state space is defined. The European and double barrier options are defined and re-expressed in a form suitable for an approximate computation of their price. The Hamiltonian, state space, and the evolution operator for the forward interest rates are discussed. European coupon bond option price is re-derived using the Hamiltonian formulation. The price of the barrier option is defined using the concept of state space and matrix elements of the evolution operator. A consistent linearization of the coupon bond function is discussed. | It shows that the constraint function for a coupon bond barrier option can be linearized. A calculation using an overcomplete set of eigenfunctions yields an approximate price for the coupon bond barrier option. The Hamiltonian formulation of the quantum field theory of forward interest rates provides a computational tool for analyzing the coupon bond European and barrier options. The computation of the coupon bond barrier option price turned out to be more difficult than the zero-coupon bond case. |
| **ID16** | Prediction Techniques | The range accrual swap in the quantum finance formulation of the Libor Market Model (LMM). | The range accrual swap is modeled in the framework of Quantum Finance and the approximate price is obtained using an expansion in the Libor volatility. The price of accrual swap is numerically analyzed by generating daily sample values of a two-dimensional Gaussian quantum field. The Monte Carlo simulation method is used to study the nonlinear domain of the model and determine the range of validity of the approximate formula. | An approximate formula was obtained by linearizing the nonlinear drift of the LMM. The simulation showed that the approximate accrual swap formula fails only for very high volatility which one does not expect for normal market conditions. The par value of the range accrual swap can be computed accurately using the approximate formula and opens the way for empirically studying the pricing of range accrual swaps. |
| **ID17** | Prediction Techniques | European options on coupon bonds are studied in a quantum field theory model of forward interest rates. | Coupon bond option price, volatility of the forward interest rates, interest rate swaps and swaptions, bond option price, perturbation expansion using Feynman diagrams, HJM limit of the approximate coupon bond option price. | The pricing of swaptions and coupon bond options using the formalism of quantum finance yields a result that is empirically better than the industry standard HJM models. The formalism of quantum finance is a flexible and transparent theoretical tool that yields accurate results for interest rate derivatives. The approximate coupon bond option price shows that the correlation between the forward prices of bonds of different maturities plays a crucial role in yielding an accurate price for the swaptions. |
| **ID18** | Prediction Techniques | Pricing formula for European caplets and coupon bond options, and American options for interest rate instruments | The payoff function for the American option is propagated backward on a time lattice using the pricing kernel and entails performing a path integral numerically. The numerical path integral is interpreted as generating the values of the American option on a tree of forward interest rates. At each step, the trial American option value obtained is compared with the payoff function. | An efficient and accurate numerical algorithm has been developed and implemented for pricing American options for interest rate instruments. The procedures are all based on the HJM model and BGM model and use variants of the binomial tree to build the tree for the interest rates and coupon bonds. The algorithm opens the way to the study of all varieties of American options, both for interest rate instruments and for correlated equity instruments as well. |
| **ID19** | Prediction Techniques | Coupon bond European and barrier options in the framework of quantum finance. | The prices of European and barrier options are analyzed by generating sample values of the forward interest rates using a two-dimensional Gaussian quantum field. The study discusses the simulation for the coupon bond European option, small volatility expansion, the simulation of the barrier option, the scaling function, the limitation of the approximate price by using linearization of the barrier, the sample white noise simulation, obtained from the basic formula for the forward interest rates. | The simulation method of quantum field theory for forward interest rates has been shown to yield efficient algorithms for pricing the coupon bond European and barrier options. The zero-coupon bond barrier option price was analyzed, and the error of the barrier was also investigated in order to reduce the error between the simulation and the exact formula. The results show that the error of the barrier can be largely removed by using a simple scaling function. Contribution - the three-factor white noise simulation can be derived from the quantum finance model of forward interest rates. |
| **ID20** | Modeling Techniques | The simulation of the Libor Market Model (LMM) in the framework of quantum finance | The numerical simulations were investigated by using the quantum finance approach, and the financial instruments (such as caplets and swaptions) for the LMM were studied using both Monte Carlo simulation and the volatility expansion. The simulation method of numerically updating Libor rates, in the framework of quantum finance, was examined and used to price interest rate instruments | The caplet price was compared with the Black’s caplet formula. It was found that the simulated caplet price fits the Black’s caplet formula quite well. Caplet price is invariant for different forward bond measures, and this important feature was confirmed in their simulation. It has provided clear evidence that the nontrivial drift is the key term in Libor Market Model, and it yields many novel properties. It shows that the simulation method is flexible and places no limitations on the parameters, it seems to be a powerful technique for pricing interest rate instruments. |
| **ID21** | Prediction Techniques | Two models, namely the bond forward interest rates, which is a linear theory and the Libor Market Model, which is a nonlinear theory. | Both the models are analyzed using Libor and Euribor data, with various approximations to match the linear and nonlinear models (interest rate correlation functions, empirical normalized propagators, stiff propagator, and market time, Euribor and Libor propagators, stochastic volatility, interest rate volatility, zero coupon yield curve and covariance, etc). | The results showed that the linear model has an accuracy of about 99% and the nonlinear model being slightly less accurate. The parameters of the stiff action turn out to be very flexible in fitting market data, and in particular allowing for a wide variation for the index. It is speculated that white noise should be replaced by the stiff action for describing all financial processes. |
| **ID22** | Prediction Techniques | Index-linked coupon bonds | The time evolution of stochastic coupons, quantum finance model of forward interest rates, correlation functions, stiff propagator, market correlators, empirical volatility, and propagators, calibration of US and Singapore models, interest rate swaptions on the US forward interest rates | A prediction for the market price of the put option for the Singapore coupon bonds was obtained. The quantum finance model was generalized to study the Malaysian case. The Malaysian forward interest rates are shown to have anomalies absent for the US and Singapore cases. The model’s prediction for a Malaysian interest rate swap was obtained. The quantum finance model for swaptions was tested using swaptions for the US Dollar and showed that the model is quite accurate. |
| **ID23** | Prediction Techniques | Hybrid intelligent method for financial time series forecasting. Novel method for forecasting financial time series. | Hybrid intelligent method for financial time series forecasting. Novel method for forecasting financial time series. A state-of-the-art hybrid algorithm, combining Dynamic Time Warping and Wavelet Transform, was presented to extract the shaped patterns in financial time series.  Adaptive-Network-Based Fuzzy Inference System (ANFIS), Particle Swarm Optimization (PSO), Quantum-behaved Particle Swarm Optimization (QPSO), Dynamic Time Warping (DTW), Wavelet Transform (WT). | Contribution - creation of a Forex trading advisory system that used both chart patterns and past exchange rate values in the decision-making process and that would perform like a real trader. It proposed a method that forecasts one-step-ahead market values by using a hybrid of ANFIS, QPSO, and WT, and extracted chart patterns by using WT and DTW at the same time. The proposed system generates trading advice instead of predicting the exact exchange rate values. The experimental results demonstrate that the proposed method performs well in financial forecasting, especially in the Forex market. Future work – to apply the proposed method to shorter timeframes, such as six hours, one hour, or even one minute, to create a real-time advisory system, or to use in other financial markets, like the stock exchange market. |
| **ID24** | Prediction Techniques | A new kernel-based similarity measure between dynamic time-varying financial networks | The study computes the average mixing matrix to summarize the time-averaged behavior of continuous-time quantum walks (CTQW) evolved on the network structures. It defines a Quantum-inspired Entropic Kernel between their quantum entropy time series through the classical dynamic time warping framework. It performs the proposed kernel on time-varying financial networks abstracted from multiple co-evolving financial time series of the New York Stock Exchange (NYSE) database. | The proposed kernel not only accommodates the complete weighted graphs through the entropy time series but also bridges the gap between graph kernels and the classical dynamic time warping framework for time series analysis. The proposed method bridges the gap between graph kernels and the classical dynamic time warping framework for multiple financial time series analysis. |
| **ID25** | Theory Applications | Classical and quantum symmetries in option pricing | Review of mathematical keywords connected with financial mathematics | Examples where there is a closed formula for the price of a European call option with a fixed interest rate. Examples of stochastic processes where Monte Carlo simulations were successfully applied. |
| **ID26** | Prediction Techniques | Quantum-inspired optimization algorithms with applications in auditing and financial data analysis | Investigation of how the subset sum problem plays a vital part in the automation of the financial auditing process and how the subset sum problem can be restated as a well-known problem architecture which can be solved by the application of gradient descent on the energy landscape of Hopfield networks. | The proposed algorithm reliably finds correct sum structures for artificial and real data. It was used to evaluate the capability of quantum annealers for the subset sum problem and it was found that for problems with a small range of values, the algorithm reliably finds correct solutions. Future challenge - to deploy existing smart auditing software to directly benefit auditors in their daily work. |
| **ID27** | Modeling Techniques | A hybrid BPNN-weighted GREY-C3LSP prediction (BWGC) | The model proposed compensation to deal with the time-varying variance in the residual errors, that is, incorporating a non-linear generalized autoregressive conditional heteroscedasticity (NGARCH) into BWGC, and quantum minimization (QM) is employed to regularize the smoothing coefficients for both BWGC and NGARCH to effectively improve model’s robustness as well as to highly balance the generalization and the localization. | The crucial problem of the overshoot and volatility clustering effects has been resolved simultaneously by a quantum minimized BWGC / NGARCH model. NGARCH can be used to deal with the inherent volatility clustering and quantum minimization is applied for regularizing NGARCH into BWGC. |
| **ID28** | Prediction Techniques | Quantum-Optimized ASVR/NGARCH composite model for time series forecasting | A weighted average between ASVR and NGARCH is realized to get the best accuracy on non-periodic short-term time series forecast | Adaptive support vector regression (ASVR) applied to the forecast of complex time series is superior to the other traditional prediction methods. The effect of volatility clustering occurring in time-series deteriorates ASVR prediction accuracy. Incorporating the nonlinear generalized autoregressive conditional heteroscedasticity (NGARCH) model into ASVR is employed for dealing with the problem of volatility clustering to best fit the forecasts. |
| **ID29** | Prediction Techniques | Time series forecast in context of the overshoot phenomenon problem and the effect of volatility clustering | Both BWGC and NGARCH models are composed linearly and then an algorithm called quantum-based minimization (QM) is particularly employed to regularize this composite model BWGC/NGARCH to best fit time series. | The study proposes incorporating a nonlinear generalized autoregressive conditional heteroscedasticity (NGARCH) into BWGC so that NGARCH is used to tackle the problem of volatility clustering effect during the time series forecast. The proposed approach can resolve the overshoot and volatility clustering effects simultaneously and outperforms the alternative models for time series forecasts. |
| **ID30** | Modeling Techniques | Qquantum-inspired cost-based feature selection for credit scoring | It adds value to credit scoring in terms of better accuracy and lower feature acquisition cost by introducing a cost-based quantum-inspired evolutionary algorithm (QIEA) feature selection method, developing a quantum-based feature selection and credit scoring framework, especially for large scale data that achieves more efficient feature selection at lower feature acquisition cost with similar or even better predictive accuracy. | Novelties – it generates a big mobile credit data set which is essential for peer-to-peer and mobile microfinancing, where limited information provided by loan applicants upfront, needs to rely on alternative data analyzing personal spending patterns, and financial and social well-being for credit risk assessment. |
| **ID31** | Prediction Techniques | Behavioral financial factors by using the pilot wave (Bohmian) model of quantum mechanics | The approach is not based on the assumption that investors act rationally and without bias and that, consequently, new information appears randomly and influences the asset price randomly. It uses methods of Bohmian mechanics to simulate the dynamics of prices in the financial market. The development of the classical Hamiltonian formalism on the price/price-change phase space is used to describe the classical-like evolution of prices. | The model expresses the complexity of the financial market: traditional description of price dynamics is completed by Schrodinger’s dynamics for the pilot wave of expectations of traders. |
| **ID32** | Market Dynamics | Mathematical modeling of price dynamics at the financial market (behavioral financial factors) | Bohmian mechanic, dynamics of prices guided by the financial pilot wave, application of the general quantum formalism to the financial market, standard deviation of price, Picard’s theorem, the problem of quadratic variation, classical and quantum financial randomness, Bohm-Vigier stochastic mechanics. | The approach can be considered as a special econophysical model in the domain of behavioral finance. It has a contribution to applications of quantum mechanics outside the microworld. |
| **ID33** | Market Dynamics | Mathematical modeling of price dynamics at the financial market (behavioral financial factors) | The model describes behavioral financial factors (e.g. expectations of traders) by using the pilot wave (Bohmian) model of quantum mechanics. The problem of the quadratic variation of the price is considered. In the Bohm–Vigier stochastic model (for the white noise fluctuations of the price velocity) is non-zero. | The model emphasizes the complexity of the financial market: the traditional description of price dynamics is completed by Schrödinger’s dynamics for the pilot wave of expectations of traders. It is a kind of socio-economic model for the financial market. |
| **ID34** | Market Dynamics | Mathematical modeling of price dynamics at the financial market (behavioral financial factors) | The Hamiltonian formalism on the price/price-change phase space describes the classical-like evolution of prices. The real trajectories of prices are determined (through the financial analog of the second Newton law) by two financial potentials: classical-like (hard market conditions) and quantum-like (behavioral market conditions). | The approach is not based on the assumption that investors act rationally and without bias and that, consequently, new information appears randomly and influences the asset price randomly. The study demonstrates that prices do not completely follow random walk. The model can be considered as a special psycho-financial model. |
| **ID35** | Risk Management | Threats of quantum computing to keep payments safe and secure | No model | No model |
| **ID36** | Modeling Techniques | Quantum versus classical models for the task of generative modeling in machine learning | The model focuses on using the Sinkhorn divergence with the Adam optimizer and its analytic gradients for the Born Machine, and loglikelihood maximization for training the Boltzmann machine. In the specific architectures for the Boltzmann and Born machines, the authors detail the underlying graph structures and the circuit Ansätze for the QCBM. Present simulated and experimental results are implemented on the Rigetti QPU using Quantum Cloud Services (QCS) to prove that the Born machine always at least matches the performance of the Boltzmann machine, and to demonstrate superior performance as the model scales. | By studying the entanglement capacity of the training Born machines, the authors demonstrate an advantage it over the Boltzmann machine. The Born machine training speed is improved by leveraging GPU-accelerated computation of the cost functions and incorporates techniques to improve running time and execution on the QPU. An alternative direction is offered, to enlarge the suite of classical model comparisons to compare the Born machine to, to solidify any perceived advantage, and extend the model into mixed states to potentially increase the expressive power. |
| **ID37** | Prediction Techniques | Piecewise linear representation of finance time series | The method is based on calculating local maxima and minima in time series. In the segmentation procedure, maxima and minima are compared with those previously found. The model involves direct diagonalization of the Hamiltonian to obtain the eigen energies. The authors explored noise reduction effects using a piecewise linear representation | It showed the possibility of having a new moving indicator based on a quantum mechanical tool. The piecewise linear representation has the advantage of being straightforward and simple. |
| **ID38** | Theory Applications | Theoretical concepts about quantum physics and classical finance | No model | No model |
| **ID39** | Prediction Techniques | Financial crash prediction | Authors have implemented the algorithm in a D-Wave quantum annealer to solve the equilibrium state of a complex financial network that predicts financial crashes. Their challenge was to find the ground state of an interacting spin Hamiltonian, which can be approximated with a quantum annealer. The size of the simulation is mainly constrained by the necessity of many physical qubits representing a logical qubit with the correct connectivity. | Their experiment paves the way for the codification of a quantitative macroeconomics problem in quantum annealers. The equilibrium configuration of a financial network before and after a perturbation with a D-Wave 2000Q quantum annealer was computed and the results were compared to alternative methods. Future challenge - to design a customized “financial quantum annealer”, a quantum processor with suitable connectivity for eliminating the problem of the limited size of the financial network. |
| **ID40** | Modeling Techniques | Quantum Least Squares Monte Carlo (Q-LSM) | The algorithm is a quantum version of the LSM algorithm and performs the error analysis considering a general set of expansion functions. The proposed algorithm is based on quantum access to a stochastic process, on quantum circuits for computing the optimal stopping times, and on quantum techniques for Monte Carlo. The proposed algorithm is based on quantum access to a stochastic process, on quantum circuits for computing the optimal stopping times, and on quantum techniques for Monte Carlo. | The authors achieved a quadratic speedup in the approximation error compared to the classical counterpart under some assumptions of smoothness on the continuation values. They deal with stochastic optimal stopping problems with a quantum advantage in the runtime. This quantum LSM can be used for problems in finance including insurance and risk management, and for many optimization problems outside finance. |
| **ID41** | Theory Applications | Gauge invariant lattice quantum field theory | The study implemented a model inspired by Ilinski, but the model is interpreted in the context of financial markets, and the numerical implementation. It is a gauge model based on the dilation group as a tool for pricing financial instruments. The financial market dynamics should be independent of their arbitrary units used for cash and assets, thus being describable using a local gauge theory which, in some sense, resembles quantum electrodynamics. | The authors see the model as an exploratory attempt because an exploration of the lattice features of the parameters has not been made. Future work may include the search for phase transitions, or spontaneous symmetry breaking. |
| **ID42** | Market Dynamics | Geometric arbitrage theory | The paper describes the intertwined dynamics of assets, term structures, and market portfolio as a constrained Lagrange system deriving it from a stochastic variational principle whose Lagrange function measures the arbitrage quantity allowed by the market. | The authors develop a conceptual structure and link arbitrage modeling in generic markets with quantum mechanics. |
| **ID43** | Risk Management | The application of quantum cryptography to financial security | A quantum blind signature scheme is proposed using χ-state and applied to the scenario of credit rating in supply chain finance. The scheme considers common attacks such as interception-and-retransmission attacks and entangled particle attacks. The four stages in a quantum blind signature scheme are concise and easy to understand. | The proposed scheme was verified that the scheme can protect the signature information. It seems to fully satisfy for requirements of blind signatures of supply chain finance. Future work will consider the design of a quantum blind signature scheme in the noise case to make the scheme more complete. |
| **ID44** | Financial Algorithms | Hybrid quantum-classical optimization model for finance | The authors describe and analyze the pruning algorithm, they introduce constraints in optimization problems (some hard constraints with an approach based on Lagrange multipliers, the variational ansatzes, and classical optimizers). An index tracking problem was used to benchmark the solutions. The algorithm iteratively discards variables through a constrained QUBO optimization process, which is driven by quantum optimization algorithms such as the QAOA subset of variables that optimizes. | The paper introduces a hybrid quantum-classical algorithm that is called heuristic k-step pruning (k-PA). This method nests two interdependent optimization problems: a classical convex optimization stage to determine the relative weights of the assets, combined with a quadratic binary optimization that determines the relevant. |
| **ID45** | Financial Algorithms | Quantum-classical algorithm for options pricing | The hybrid quantum-classical algorithm is meant to solve the Schrödinger equation, which by simple transformations is equivalent to the PDE satisfied by the option price. The approach is based on the equivalence between the pricing partial differential equation and the Schrödinger equation in imaginary time. The strategy is to build a shallow quantum circuit approximation to this equation, only requiring a few qubits. It solves the Black-Scholes PDE by first transforming it into the Heat Equation and then converting the option price to a Quantum-State which is effectively a wave function. The wave function is solved by the Hybrid Quantum and Classical Algorithm where a Quantum Circuit of imaginary time evolution is built with the help of McLachlan’s invariance Principle. | The algorithm originated from quantum chemistry, it focuses on pricing European and Asian options in the Black-Scholes model. A new route to investigate the use of quantum techniques in quantitative finance is proposed. This constitutes a promising candidate for the application of quantum computing techniques (with a large number of qubits affected by noise) in quantitative finance. The challenge is the requirement for an ansatz circuit and the corresponding solution of an optimization problem. More work is needed in the future to design an efficient ansatz for more complex financial products, or in the development of an ansatz-free approach. |
| **ID46** | Market Dynamics | An efficient econophysics’ proxy for stock exchange explosion | The paper tries to predict the Reynolds number for the future and to trace back the behavioral links. The authors check for the presence of martingale in the Reynolds number in Nifty. Financial Reynolds number it seen as a significant indicator for investors to exit the market prior to a catastrophe. | Though the frequency of financial Reynolds number in the vicinity of 10, steadily decreased over the years, it would be prudent for the investors to stay away from the bourses during such a period. On the other hand, whenever it tends to zero or near zero, the volatility will be extremely low, hence it would provide a safe zone for trading. Policy makers too could use the financial Reynolds number as an indicator of market volatility and treat any value closer to or above 10 to be significantly volatile zone, thus cautioning the investors well in advance. |
| **ID47** | Risk Management | Quantum computing in derivative pricing and risk estimation | It is a conceptual review of the state of the art and recent advances in quantum computing applied to derivative pricing and the computation of risk estimators like Value at Risk. The authors first review the main models and numerical techniques employed to assess their value and risk on classical computers. Then, they describe some of the most popular quantum algorithms for pricing and VaR. | Future work may target loading a probability distribution (the direct benefits in this matter go to VaR problems), generating a probability distribution (this would be the direct equivalent of simulating a stochastic differential equation), and loading a payoff function (this case is only relevant for pricing). |
| **ID48** | Risk Management | Quantum game theory approaches financial volatility risk. | The model translates into a quantum repeated game setting that constitutes Mandelbrot’s hypothesis of economic complexity with financial market efficiency, such that turbulence and multifractal signatures do not come from anomalous trading behavior or speculative trading systems but from the business cycle nonlinear dynamics. | The model incorporates the business cycle by adapting the standard economic tradition, in business cycle dynamics modeling extending to the quantum setting a standard harmonic oscillator model of the business cycle, within the context of an adaptive quantum business optimization problem with nonlinear evolutionary conditions. The model provides a quantum game theoretical approach to market turbulence with chaotic intrinsic time leading to multifractal signatures in volatility dynamics. |
| **ID49** | Financial Algorithms | Two quantum optimization algorithms applied to portfolios of trade finance portfolios | The method used is to map the financial risk and returns for a trade finance portfolio to an optimization function of a quantum algorithm developed in a Qiskit tutorial. The Variational Quantum Eigensolver (VQE) is used for optimization applications harnessing energy states to calculate the function of the variables it needs to optimize. In financial services, the VQE has been used in stock portfolio optimization in the tutorial. The Quantum Approximate Optimization Algorithm (QAOA) is a hybrid quantum-classical variational algorithm designed to address combinatorial optimization problems. | The results show that whilst there is no advantage seen by using the quantum algorithms, the performance of the quantum algorithms has no statistically significant degradation. Therefore, it is promising that in the future, with expected improvements in quantum hardware, the theoretically superior processing speeds, and data volumes that quantum offers, will also be applicable to trade finance. |
| **ID50** | Theory Applications | Quantum game theory approach to financial market stability | Through the extension of the well-known hawk–dove game by a quantum approach, authors showed that dependent on entanglement, evolutionary stable strategies also can emerge, which are not predicted by the classical evolutionary game theory and where the total economic population uses a non-aggressive quantum strategy. A model was developed, it is based on this game type and comprises the relevant parts of the behavior of these constructors and sellers to mirror the starting conditions of the financial crisis. | Evolutionary game theory predicts that under the condition of strategic dependence, a certain degree of aggressive behavior remains within a given population of agents. |
| **ID51** | Financial Algorithms | Quantum computing for financial applications | The paper presents a summary of the state of the art of quantum computing for financial applications, with particular emphasis on stochastic modeling, optimization, and machine learning, describing how these solutions, adapted to work on a quantum computer, can potentially help to solve financial problems, such as derivative pricing, risk modeling, portfolio optimization, natural language processing, and fraud detection, more efficiently and accurately. | The authors’ conclusion was that the quantum revolution is still at the beginning, potential for quantum technology to transform the financial industry is observed. The community has developed potential quantum solutions for portfolio optimization, derivatives pricing, risk modeling, and several problems in the realm of artificial intelligence and machine learning. |
| **ID52** | Theory Applications | Finance and the quantum mechanical formalism | The paper deals with the Bohmian mechanics approach and the path integration approach | Limits - applications of quantum field theory to finance were not discussed. |
| **ID53** | Financial Algorithms | Hybrid quantum-classical neural network for classification | Exploration of quantum machine learning for business applications (e.g. in finance), | Hybrid Quantum Network for classification of finance and MNIST data |
| **ID54** | Modeling Techniques | It presents an agent behavior-based microscopic model that induces jumps, spikes, and high volatility phases in the price process of a traded asset. | Using the mathematical framework of Henkel, the authors set a finite network of heterogeneous agents interacting in continuous time. The agent’s behavior is thereby inspired by the dynamics of excited particles in a quantum system. They linked the endogenous dynamics to an asset price process by specifying agents' individual trading propensity and excess demand functions together with an overall pricing rule. | It shows the conditions under which the average agent excitement as well as the price process converge to a diffusion process when the number of market participants tends to infinity. Limitation - the missing feedback of the price process on the endogenous dynamics as well as the strong Markov property of the model seems unrealistic. |
| **ID55** | Market Dynamics | Evolution of financial risk management | No model | No model |
| **ID56** | Modeling Techniques | Hybrid classical quantum computing in financial options modeling | Hybrid computing, ASA Algorithm, Path-Integral Algorithm, Direct Kernel Evaluation, Monté Carlo vs Kernels, Quantum Path Integral Algorithms | The authors demonstrate how some hybrid classical-quantum systems may be calculated quite well using only classical (super-)computers. The quantum version can be used for many quantum systems, which are becoming increasingly important as experimental data is increasing at a rapid pace for many quantum systems. |
| **ID57** | Financial Algorithms | Quantum Monte Carlo integration with pseudorandom numbers | The use of PRNs, which the authors originally proposed in the context of the quantum algorithm for Monte Carlo, is the key factor also in this paper since it enables parallel computation of the separable terms in the integrand. Furthermore, they pick up one use case of this method in finance, the credit portfolio risk measurement and estimate to what extent the complexity is reduced. | The number of such repeated operations can be reduced by a combination of the nested QAE and the use of pseudorandom numbers (PRNs) if the integrand has a separable form with respect to contributions from distinct random numbers. Future work must explore the possibility of utilizing such a feature in other ways and make a quantum algorithm for Monte Carlo more efficient. |
| **ID58** | Theory Applications | Quantum-like models for financial processes | The authors analyze the complexity of financial processes by comparing classical and quantum-like models for randomness. A quantum-like probabilistic description is more natural for financial markets than the classical one. It studied the possibility of application of the quantum probabilistic model to agents of the financial market. | Quantum-like probabilistic behavior is a consequence of the context of statistical data in finances. There is not any classical stochastic process that will match with the real financial data, because there is not a single Kolmogorov space describing the whole financial market. Authors show that, although the direct quantum (physical) reduction (based on using the scales of quantum mechanics) is meaningless, one may apply so-called quantum-like models. |
| **ID59** | Theory Applications | Potential financial applications of quantum probability (QP) | The model serves as a generalization to the classical probability (CP) scheme and relaxes the core axioms of commutativity and distributivity of events. The agents form subjective beliefs via the rules of projective probability calculus and make decisions between prospects or lotteries by employing utility functions and some additional parameters given by a so-called ‘comparison operator. The QP model is based on subjective expected utility and defines the core mathematical rules pertaining to lottery selection from an agent’s (indefinite) comparison state. | The main motivation for the application of the QP mathematical framework as a mechanism of probability calculus under non-neutral ambiguity attitudes among agents coupled with a state dependence of their utility perception derived from its ability to generalize the rules of classical probability theory and capture the indeterminacy state. |
| **ID60** | Market Dynamics | Dynamics of propagator in financial derivates | Authors study the sensitivity of the implied volatility of caplets and swaptions according to the three dominant dynamics of the propagator, and the change of the zero-coupon bond option price according to the two dominant dynamics of the integrated propagator. They reviewed the quantum finance model and how to price interest rate derivatives using volatility expansion. | The PCA in the impact of the propagator shows three important innovations, the level-like, the skew-like, and the smilelike impact. The implied volatility of caplets shows the parallel shift influenced by the level-like impact and the skewed movement for the short time to maturity influenced by the skew-like impact. The smile-like impact of the propagator makes the direction of implied volatility shift arbitrary with respect to time to maturity. Future work has to use sensitivity analysis to develop a new hedge method that complements the previous hedge technique. |
| **ID61** | Prediction Techniques | Enhancing quantum machine learning performance with linear discriminant analysis | In the paper different preprocessing methods are compared and classified as methods on small and larger datasets with a binary target. The objective is to determine a specific architecture for preprocessing, dimensionality reduction of the dataset structure, the encoding manner, and the corresponding classifier. | This study shows that we can achieve better classical encoding and performance of quantum classifiers by using Linear Discriminant Analysis (LDA) during the data preprocessing step. As a result, the Variational Quantum Algorithm (VQA) shows a gain of performance in balanced accuracy with the LDA technique and outperforms baseline classical classifiers. |
| **ID62** | Prediction Techniques | Price prediction with PCA-machine learning hybrid model (Principal Component Analysis) | The suggested model employs feature engineering to generate the financial and technical analysis time series data and uses the trend deterministic data preparation layer to turn the data into a discrete form. The principal component analysis (PCA) method is applied to discrete-form data to introduce the dimensionality of a data set with numerous interrelated qualities while keeping the maximum variability in the data set. The accuracy of the tailored PCA-ML forecasting model is evaluated using classification metrics. | A PCA-machine learning (ML) hybrid forecasting model was proposed. The experimental findings suggest that the technical factors are signified as trend signals and that the PCA approach combined with ML models outperforms the comparative models in prediction performance. When discrete/trend data is integrated with PCA-ML models, the performance of the models improves significantly, according to experiments. |
| **ID63** | Modeling Techniques | Quantum principal component analysis for pricing interest-rate financial derivatives | The model considers several noisy components to accurately describe the dynamics of several time-maturing forward rates, which can be gathered in a cross-correlation matrix. The eigenvectors corresponding to the largest eigenvalues of this matrix provide the principal components of the correlations. | This manuscript is the first step toward the design of a general quantum algorithm to fully simulate on quantum computers the Heath-Jarrow-Morton model for pricing interest-rate financial derivatives. It shows that practical applications of quantum computers in finance will be achievable soon. |
| **ID64** | Modeling Techniques | Optimum portfolios | Market dynamics are discussed as a process of continuous observation of market state variables. The assumption is that the market state corresponds to a pure quantum state. | The price is considered a new quantum variable. The model derives a quantum analog of the Black-Scholes formula for the price of financial variables in the assumption that the market dynamics can be considered as its continuous weak measurement at no-arbitrage condition. |
| **ID65** | Theory Applications | The prediction of financial crashes as well as dynamic portfolio optimization | Authors describe an algorithm to find the stable state of a financial network using a present-day quantum computer. This result can be used for financial crash prediction. The paper contains some comments on the different types of quantum strategies, such as those based on quantum annealers, universal gate-based quantum processors, and quantum-inspired Tensor Networks. | The study contains two basic applications of quantum optimization for financial problems, namely, financial crash prediction, and dynamic portfolio optimization. These examples show that real business value can be derived from present-day quantum computers. This is particularly true for the portfolio optimization case, where we found the best investment portfolio by optimizing over 52 assets and four years of data. |
| **ID66** | Financial Algorithms | An algorithm called Approximate Amplitude Encoding (AAE) was proposed. AAE is a data loading algorithm that works with fewer gates, despite the unavoidable error caused by the limited representation ability of a fixed ansatz and possibly the incomplete optimization | The key of the algorithm is to variationally train a shallow parameterized quantum circuit, using the results of two types of measurement. The standard computational-basis measurement plus the measurement in the Hadamard-transformed basis was introduced to handle the sign of the data components. The variational algorithm changes the circuit parameters to minimize the sum of two costs corresponding to that two-measurement basis, both of which are given by the efficiently computable maximum mean discrepancy. | It can effectively load all the components of a given real-valued data vector into the amplitude of the quantum state, while the previous proposal can only load the absolute values of those components. The algorithm realizes the loading of a time series of real stock prices on a quantum state with a small approximation error, and thereby it enables the construction of an indicator of the financial market based on the stock prices. The combination of the AAE algorithm and the variational quantum Singular Value Decomposition (qSVD) algorithm offers a new quantum algorithm for computing the SVD entropy for stock price dynamics. |
| **ID67** | Theory Applications | Non-relativistic gauge/gravity correspondence for scale-invariant deformations | The authors study scale-invariant, but not necessarily conformal invariant deformations of non-relativistic conformal field theories from the dual gravity viewpoint. They present the corresponding metric that solves the Einstein equation coupled with a massive vector field. They found that when the Galilean invariance is assumed, the scale-invariant deformation always preserves the non-relativistic conformal invariance. | A novel application of non-relativistic gauge/gravity correspondence is proposed. One is the Reggeon field theory, and another is the non-linear quantum finance. There is a strongly coupled regime of both theories that is beyond the scope of the perturbative field theories, and the dual gravity computation is promising. Future work has to focus on deriving the phenomenological non-relativistic background from the AdS/QCD correspondence whose theoretical foundation is much firmer. |
| **ID68** | Theory Applications | The quantum-like dynamics of financial markets in terms of a non-differentiable price–time continuum having fractal properties. | The statistical scaling, the non-differentiability hypothesis, and the equations of motion. | Authors suggest that the wave function amplitude is a measurable quantity in the sense that it can be directly estimated from price data. Through it, we can get in principle the mathematical description of leading market factors. Studying wave function evolution might help in solving the problem of price predictability. |
| **ID69** | Theory Applications | The probability distribution function (PDF) for prices on financial markets | It is shown how on that basis the quantum-like description for financial markets arises and different financial market models are mapped by quantum mechanical ones. | It is obvious for further research that PDF-specific features could be modeled by variation of potential function form. The novelty is that it results in analytically solvable equations, which invites empirical investigations using widely available financial data. |
| **ID70** | Modeling Techniques | Quantum computation applied to financial problems | The paper reviewed quantum optimization algorithms and exposed how quantum annealers can be used to optimize portfolios, find arbitrage opportunities, and perform credit scoring. The paper highlights different fields within finance that could benefit from a computational speedup using quantum computers. | This field is developing at a striking rate, partly due to experimental developments in quantum hardware, which are surpassing all expectations, and partly due to conceptual leaps, which promise gigantic speedups for widely applicable algorithms. Future work may integrate how quantum technologies can be relevant to the blockchain and cryptocurrencies, or discuss quantum finance, quantum money, the impact of quantum cryptography in the security of financial transactions, and the applications of quantum simulators in finance. |
| **ID71** | Financial Algorithms | Forecasting financial crashes with quantum computing | The authors show that the problem of predicting financial crashes and assessing the equilibrium of a financial network, is amenable to quantum annealers, at least for simple financial toy models. The authors map the equilibrium condition of a toy-model financial network to the ground-state problem of a spin-1/2 quantum Hamiltonian with 2-body interactions, i.e., a quadratic unconstrained binary optimization (QUBO) problem. The equilibrium market values of institutions after a sudden shock to the network can then be calculated via adiabatic quantum computation and, more generically, by quantum annealers. | The result shows that quantum computers could help in forecasting in addition to other known applications in finance. Further research could explore ways of improving the efficiency and accuracy of the procedure. More complex financial network models may require extra resources which were not considered by authors. |
| **ID72** | Modeling Techniques | Enterprise financial early warning model | Quantum Rotation Gate is used to optimize four algorithms, namely, Fruit Fly Optimization Algorithm (QFOA), Bee Colony Optimization Algorithm (QABC), Particle Swarm Optimization (QPSO), and Ant Colony Optimization (QACO). | The authors found that the Quantum Fruit Fly Optimization Algorithm and the optimized SVR Financial Early Warning Model are superior to the other three algorithms in convergence speed, optimization stability, and financial early warning stability. In addition, it is found that the four models are very close to each other in terms of convergence rate, model stability, and prediction error. The Quantum Rotation Gate is superior in the ability to optimize the four algorithms. It is suggested that the Fruit Fly Optimization Algorithm can be used to optimize SVR to construct a Financial Early Warning Model in the future. Future work may focus on chaos theory or wavelet theory. |
| **ID73** | Prediction Techniques | Hybrid deep quantum neural network for financial predictions | The QuantumLeap system consists of an encoder that transforms a partitioned financial time series into a sequence of density matrices; a deep quantum network that predicts the density matrix later; and a classical network that measures the maximum price reached by the security at a later time, from the output density matrix. The deep quantum network is isomorphic to a deep classical neural network and is computationally tractable in terms of the number of hidden layers. | The paper presents a new hybrid deep quantum neural network for the prediction of financial trajectories. This system consists of a deep quantum neural network for quantum prediction and a deep classical neural network, which acts as a measurement apparatus that extracts security prices from predicted quantum density matrices. The deep quantum network employs a new quantization scheme for financial time series and is computationally tractable in terms of the number of hidden layers |
| **ID74** | Risk Management | Assessing the quantum-resistant cryptographic agility of routing and switching IT network infrastructure in a large-size financial organization | The study investigates quantum-resistance cryptography from the hardware perspectives of routing and switching technologies using diffusion of innovation theory. The study integrates enterprise governance to meet the challenges presented by quantum computing with a focus on cryptographic agility. | The research study was limited to the top three data center routing and switching vendors, further research may expand the vendor base. The research did not cover such technologies as identity management platforms, secure web gateways, application delivery controllers, and firewalls as well as intrusion prevention and detection platforms. Another limitation of this study is related to enterprise software applications. Quantum computing will impact all aspects of IT including enterprise applications. Further research can assess the cryptographic agility of enterprise-level applications. |
| **ID75** | Theory Applications | Review of development in quantum game theory and possible application in economics and finance. The review is addressed to non–non-specialists. | No model | No model |
| **ID76** | Risk Management | Quantum-like approach to financial risk: quantum anthropic principle | The authors show that it is possible to define a risk inclination operator acting in some Hilbert space that has a lot in common with the quantum description of the harmonic oscillator. The approach has roots in the recently developed quantum game theory and quantum computing. | The formalism of quantum theory may provide us with tools of unexpected power that combined with methods of game theory may allow for a much deeper understanding of financial phenomena than it is usually expected. |
| **ID77** | Prediction Techniques | The paper presents state-of-the-art quantum algorithms for financial applications, with a particular focus on those use cases that can be solved via Machine Learning. | The paper contains an introduction to quantum ML techniques and their applications in the financial services sector. There are seven machine learning tasks, for which several quantum algorithms have been previously proposed in the literature: regression, classification, clustering, generative learning, feature extraction, sequential decision-making, and Natural Language Processing. | The realities of implementing quantum computing techniques in the financial sector must face challenges imposed by hardware limitations. |
| **ID78** | Modeling Techniques | Quantum finance-based hybrid deep reinforcement learning portfolio investment system | The paper introduces an intelligent portfolio investment system based on the integration of DRL, Quantum Finance Theory (QFT). It consists of two agents: 1) A trading agent based on a Deep Deterministic Policy Gradient (DDPG) algorithm to generate continuous actions for investment weighting: and 2) A risk-control agent based on a Policy Gradient (PG) algorithm that produces discrete actions according to each day’s Quantum Price Levels (QPLs). One significant merit of integrating two intelligent agents is that they can cooperate to make more reasonable and stable fund distribution adjustments in the portfolio investment. | The system has better performance over a range of previous methods in the test dataset and unseen dataset. Currently, the QPL-inspired risk control mechanism would do the same operation on each product in the portfolio when a specific close decision is made. Limitation - the portfolio value is calculated by allowing all products to do the same operation, but in real-life situations, it is more appropriate to allow operations of each product in the portfolio to be separate. Future work - multi-agent mechanism. |
| **ID79** | Theory Applications | Time independent pricing of options in range-bound markets | No model | No model |
| **ID80** | Modeling Techniques | Quantum algorithm for the Monte Carlo pricing of financial derivatives | The study assumed that the distribution of the underlying random variables, i.e., the martingale measure, is known and the corresponding quantum states can be prepared efficiently. | It shows how the relevant probability distributions can be prepared in quantum superposition, the payoff functions can be implemented via quantum circuits, and the price of financial derivatives can be extracted via quantum measurements. The amplitude estimation algorithm can be applied to achieve a quadratic quantum speedup in the number of steps required to obtain an estimate for the price with high confidence. Investigating the promising advantages of the continuous variable setting in a financial context in more detail will be left for future work. |
| **ID81** | Theory Applications | Quantum finance symmetries | It is shown that Black-Scholes equation is invariant under Schrödinger group. To do this, the one-dimensional free non-relativistic particle and its symmetries are revisited. To get the Black-Scholes equation symmetries, the particle mass is identified as the inverse of square of the volatility. | The conclusion is that physical techniques can be employed to study other disciplines. The paper contains a theoretical approach. |
| **ID82** | Theory Applications | Quantum finance symmetries | The one-dimensional free non-relativistic particle and its symmetries are revised and the particle mass is identified as the inverse of the square of the volatility. A Schrödinger algebra representation is constructed using financial variables. | The paper contains a theoretical approach. |
| **ID83** | Market Dynamics | Quantum coupled-wave theory of price formation in financial markets | In this model bid and ask prices are represented by eigenvalues of a 2x2 price operator corresponding to ‘bid’ and ‘ask’ eigenstates, while randomness of price operator results in price fluctuations that destroy oscillatory effects. Security prices behave as quantum-chaotic quantities, not classical-chaotic. The authors discuss the ergodicity properties of price formation and show how directional price movement occurs due to ergodicity violation in a quantum process instead of the commonly believed forces acting on price. | The authors develop a theory of bid and ask price dynamics in which the two prices form due to quantum-chaotic interaction between buy and sell orders. Quantum framework overcomes the default assumptions of stochastic framework about unlimited liquidity and price availability and allows to model processes where execution is a strong factor. The coupled-wave model is the simplest of quantum models that deal with bid and ask prices as different yet connected variables. |
| **ID84** | Market Dynamics | Quantum coupled-wave theory of price formation in financial markets | Quantum theory is used to model secondary financial markets (A Hilbert Space Representation of the Market, Moving Cash, Creating and Destroying Securities, Temporal Market Evolution, Cash Flow, Trading Securities, The Evolution of Stock Prices in an Equilibrium Market, etc.). | The quantum models presented in the paper are not meant to be overly realistic, but perhaps reflect some generic aspects of quantum finance. The paper contains a theoretical approach to quantum finance. |
| **ID85** | Theory Applications | Determinants of finance acquisition by tech startups over the lifecycle. This study investigates the factors that enable a tech startup to obtain finance over its lifecycle based on data gathered through a semi-structured questionnaire and in-depth interviews with the founders/CEOs of 93 tech startups in Bangalore. | No model | No model |
| **ID86** | Risk Management | A quantum algorithm to compute the market risk of financial derivatives | The authors show that employing quantum gradient estimation algorithms can deliver a further quadratic advantage in the number of the associated market sensitivities, usually called greeks. By numerically simulating the quantum gradient estimation algorithms on financial derivatives of practical interest, we demonstrate that not only can we successfully estimate the Greeks in the examples studied, but that the resource requirements can be significantly lower in practice than what is expected by theoretical complexity bounds. | Contributions: numerically study of the quantum gradient estimation algorithms for functions of practical interest to financial market risk, a method to construct a second-order accurate oracle for quantum gradient estimation for functions computed using quantum amplitude estimation, a way to improve gradient estimation algorithms using classical maximum likelihood estimation (MLE), a technique to employ automatic differentiation (AD) methods on quantum computers which can enhance the quantum gradient estimation performance in certain cases, the resource estimates for quantum advantage in financial derivative pricing from prior research. |
| **ID87** | Modeling Techniques | Quantum-behaved Particle Swarm Optimization (QPSO) Algorithm to solve multi-stage portfolio optimization problem | The objective function is the classical return-variance function. The performance of the algorithm is demonstrated by optimizing the allocation of cash and various stocks. Experiments are conducted to compare the performance of the portfolios optimized by different objective functions with the Particle Swarm Optimization (PSO) algorithm and Genetic Algorithm (GA) in terms of efficient frontiers. | Compared with PSO and GA, QPSO generates better efficient frontiers with better objective function value and robustness. Furthermore, the convergence rates of the algorithms were studied and the results show that QPSO could converge to the optima rapidly, while PSO may encounter premature convergence and GA may not reach the optima due to its slow convergence rate. It is suggested that QPSO is a promising solver for multistage stochastic financial optimization problems. |
| **ID88** | Theory Applications | Unveiling the Quantum Potential in Financial Markets | The authors model real markets by the Bohmian quantum approach bearing a quantum potential that guides the price return fluctuations. It is shown that this quantum potential confines the price returns of real markets in a scale-invariant manner, which proves to be different for emerging and efficient markets. | It is shown that the quantum potential provides boundaries that confine the price return fluctuations within their walls. In other words, by increasing the potential, the possibility of a price return decreases. Implementing the quantum potential would enable us to sketch a robust pattern for the price return fluctuations of a financial market. |
| **ID89** | Theory Applications | Quantum computing in finance: revolutionizing financial applications | It is a review of quantum optimization. Quantum adiabatic computations are adapted to solve financial problems. | The authors summarize the quantum stochastic modeling techniques, particularly the quantum Monte Carlo method, which is useful in derivative pricing and risk assessment. They discuss that quantum machine learning is expected to boost financial big data analysis, where the training efficiency of the model is significantly better than that of the classical model, making it more suitable to meet the need for financial institutions to offer big new data-driven services aligning with diverse consumer behavior. |
| **ID90** | Prediction Techniques | Financial Time Series Prediction using PEEMD-QNN | It is proposed a new model, called primary ensemble empirical mode decomposition combined with a quantum neural network (PEEMD-QNN) in predicting the stock index. PEEMD-QNN takes advantage of the PEEMD which retains the main component of modal component and QNN. The model uses a three-layer network structure, four input layer quantum neurons, three hidden layer quantum neurons, and one output layer quantum neuron. | The proposed PEEMD-QNN model has higher accuracy than the BP neural network, QNN model, and EMD-QNN model in stock market prediction. The innovation is to provide a prediction model to get improved prediction accuracy. |
| **ID91** | Risk Management | Feasibility of quantum risk measurement in financial institutions | In a typical market risk application that makes use of simulating future market scenarios, such as a Monte Carlo-based Value-at-Risk (VaR), many risk factors and their joint evolution need to be reflected. Furthermore, the corresponding changes in the present value (PV) of instruments in a portfolio require revaluations in each potential future state. The situation in a counterparty credit risk context is similar: scenarios across a multitude of risk factors and, additionally, numerous time steps need to be combined with portfolio revaluations. | While conceptual solutions and small-scale circuits are feasible, the leap needed for real-life applications is still significant. The contribution of the study is an exploration of the feasibility of using quantum computing in actual large-scale risk applications in financial institutions. Practical applications in quantitative finance are still in their infancy. |
| **ID92** | Modeling Techniques | Optimal quantum control for stochastic systems in finance | The objective is to design an optimal control for a class of quantum stochastic systems in which the state is governed by a Quantum Stochastic Differential Equation (QSDE). In this dynamical equation, the Brownian motion is modeled as a quantum process. In other words, the disturbance of the system is modeled by a quantum process with random variables that satisfy the Schrödinger equation. It is shown that the quantum process is a Brownian motion. | A theorem for guaranteeing the existence and uniqueness of solutions to the QSDES is proved. Additionally, a new optimal stochastic control problem is formulated, and based on the necessary optimality conditions, an optimal quantum control law is designed explicitly. Four theorems and two lemmas, for facilitating the optimal controller design algorithm, are proved. Finally, to demonstrate the applicable results, two financial problems, Merton portfolio allocation, and optimal pairs trading problem are simulated by using the presented method. As the simulation results indicate, portfolio optimal performances, minimum risk, and maximum return are achieved via the presented method. |
| **ID93** | Modeling Techniques | Operator-valued quantum stochastic optimal control theory | The stochastic LQR optimal problem in Fock space was investigated in quantum formalism, based on the quantum stochastic calculus. For this purpose, system dynamics was described by Hudson–Parthasarathy type Quantum Stochastic Differential Equation (QSDE) in Fock space, and by associating a quadratic performance criterion with the QSDE, the Quantum Stochastic Linear Quadratic Regulator (QS-LQR) control problem was modeled and then solved by considering the new corresponding Hamilton–Jacobi–Bellman equation. | The optimal quantum pairs trading strategy was obtained and based on this strategy; a portfolio was constructed. The obtained portfolio has low risk and high return. |
| **ID94** | Theory Applications | Transformation of Black–Scholes equation in option pricing theory | The Black–Scholes equation of the option pricing theory to minimize the risk through the stocks is studied. The solutions are obtained in terms of exceptional Laguerre polynomials. Moreover, higher-order supersymmetric representations are studied with a special case of third order. The Darboux transformation of the heat equation linked to the Black–Scholes system is given, and a new potential is shown. | The authors gave the solutions of the Black–Scholes equation in terms of exceptional orthogonal polynomials and obtained a potential model using higher-order supersymmetric quantum mechanics. Moreover, they have given the transformation of the Black–Scholes equation into the heat equation with solutions. Using third order Darboux transformations, a singular potential model is obtained while the initial potential was a constant. Finally, it is noted that one can obtain Hermite polynomial solutions of the heat equation derived from the Black–Scholes equation and the time parameter can be varied with a parameter of the Gaussian wavefunction. |
